# Impact of precursor-derived peracetic acid on post-weaning diarrhea, intestinal microbiota, and predicted microbial functional genes in weaned pigs

**DOI:** 10.3389/fmicb.2024.1356538

**Published:** 2024-01-25

**Authors:** Salvatore Galgano, Leah Conway, Adrian Fellows, Jos Houdijk

**Affiliations:** ^1^Monogastric Science Research Centre, Scotland's Rural College (SRUC), Edinburgh, United Kingdom; ^2^Aga2Tech Ltd, Halifax, United Kingdom

**Keywords:** antimicrobial alternative, metagenome, microbiota, ortholog, peracetic acid, pig, zinc oxide

## Abstract

Post-weaning diarrhea affects piglets in the nursery phase of production, leading to a substantial impact both at the farm and financial levels. The multifactorial etiology of this disease includes housing conditions, pig genetics, microbial composition, and metagenomic assets. Among the common therapeutic approaches, the widely used zinc oxide underwent a European Union ban in 2022 due to its negative environmental impact and correlation to increased antimicrobial resistance. During this study, we have tested two levels of inclusion of the potential antimicrobial alternative peracetic acid, delivered in water via the hydrolysis of the precursors sodium percarbonate and tetraacetylethylenediamine, in comparison to zinc oxide and an untreated control during a 2-week animal study. We assessed the microbial composition and predicted the metagenome, together with performance and physiological parameters, in order to describe the microbial functional role in etiopathology. Both zinc oxide and peracetic acid resulted in amelioration of the diarrheal status by the end of the trial period, with noticeable zinc oxide effects visible from the first week. This was accompanied by improved performance when compared to the first-week figures and a decreased stomach pH in both peracetic acid levels. A significant reduction in both stomach and caecal Proteobacteria was recorded in the zinc oxide group, and a significant reduction of *Campylobacter* in the stomach was reported for both zinc oxide and one of the peracetic acid concentrations. Among other functional differences, we found that the predicted ortholog for the zonula occludens toxin, a virulence factor present in pathogens like *Escherichia coli* and *Campylobacter jejuni*, was less abundant in the stomach of treated pigs compared to the control group. In water, peracetic acid delivered via precursor hydrolysis has the potential to be a valid intervention, an alternative to antimicrobial, to assist the weaning of piglets. Our findings support the view that post-weaning diarrhea is a complex multifactorial disease with an important metagenomic component characterized by the differential abundance of specific predicted orthologs and microbial genera in the stomach and caecum of pigs.

## 1 Introduction

Pigs account for 50% of the total meat production in the European Union (Popescu, 2020), totaling 110 million tons produced worldwide in 2020 (Mickiewicz, 2022). As part of pork production, weaning is characterized by a diet switch from liquid to solid and concurrent physiological and microbiological changes that negatively impact the growth rate, at least temporarily, and that can lead to post-weaning diarrhea (PWD) (Madec et al., 2000). PWD is usually associated with important financial implications and with a pivotal role in the spread of antimicrobial resistance (AMR) (Laine et al., 2008). Although PWD is a multi-factorial gastrointestinal disease, enterotoxigenic *Escherichia coli* has been identified for its main role in the pathogenesis (Laine et al., 2008). The abrupt change from sow milk to plant-based commercial feed greatly affects the porcine gut microbial communities (Guevarra et al., 2019). Indeed, a considerable factor in shaping the early microbial populations is the maternal contribution, via both feed and gestation (Jiang et al., 2019). Firmicutes and Proteobacteria are predominant in the ileal digesta of 2-day-old piglets (Konstantinov et al., 2006), and *Clostridium* and *Escherichia–Shigella* are predominant at day 1, whose abundance decreases toward day 20, whereas *Bacteroides* and *Lactobacillus* increase (Chen et al., 2018), thus *Prevotella* and *Clostridium* abundance increase soon after weaning (Pajarillo et al., 2014).

The impact of cost due to therapies, slower growth, and increased mortality associated with PWD underlies the importance of finding sustainable alternatives (Bonetti et al., 2021). Among the common therapeutic approaches, colistin has been commonly used in the past due to its high efficacy against enteric diseases (Ahmed et al., 2021); however, plasmid-mediated colistin resistance genes have been reported in PWD pig isolates (Curcio et al., 2017). Within the non-antibiotic molecules, zinc oxide (ZnO) has been extensively used to alleviate porcine PWD (Ou et al., 2007); nevertheless, ZnO faced a European Union ban in 2022 due to its deleterious environmental implications (Ekhlas et al., 2023).

Peracetic acid (PAA) is a wide-spectrum biocide used in several contexts (Kitis, 2004), whose impacts on microbiota and performance *in vivo* via precursor hydrolysis have been demonstrated in poultry, both in water (Galgano et al., 2023b) and in feed (Galgano et al., 2023a). During this study, we administered PAA in water to weaning pigs via the hydrolysis of the precursors sodium percarbonate (SP) and tetraacetylethylenediamine (TAED). In particular, we tested the effects of the two levels of inclusion, 50 and 150 mg/kg, in comparison to in-feed supra-nutritional ZnO at 3,100 mg/kg, and to an untreated control on a total of 48 piglets in 24 pens. We assessed the fecal scoring (FS) as a measure of the diarrhetic status, performance parameters, microbial composition, and predicted ortholog metagenome in three gut locations. We were able to identify a treatment-driven phenotype connected to ameliorated PWD symptoms and correlate this to a possible microbial functional role, contributing to the advancement of the knowledge characterizing the etiopathogenesis of PWD as a multifactorial disease.

The hydrolysis-mediated PAA delivery method does not result in the generation of harmful by-products, instead leading to the final formation of H_2_O and CO_2_, and therefore, the broad antimicrobial alternative, in-water PAA, could be a solution to one of the most economically relevant diseases in pig husbandry, contributing to lowering down the abundance of antimicrobial resistant genes associated with the pig industry.

## 2 Materials and methods

### 2.1 Animal study, sample collection, DNA isolation, and pH measurement

The 14-day animal study was carried out at the SRUC Easter Howgate farm, following SRUC ethical approval (PIG AE 20-2021). A total of 48 piglets were selected from the farm, from five litters, at weaning and allocated to 24-floor pens with two pigs per floor pen in six rooms (i.e., four pens per room). Piglets at placement were balanced for sex, initial body weight, and litter origin per treatment as much as possible, while temperature and humidity were automatically controlled throughout the trial. The pens were allocated to four treatments (Table 1), with six replicate pens (i.e., 12 pigs) per treatment, and each pen was provided with litter (wooden shavings), enrichment material (e.g., boots), and a porch-covered area, complying with welfare standards. Supra-nutritional 3,100 mg/kg of ZnO was added to the feed at the mill (Target Feed Ltd., Whitchurch, UK), while water treatment was prepared daily on the farm, as previously described (Galgano et al., 2023b), via mixing an appropriate quantity of precursors (TAED and SP) to achieve either 50 or 150 mg/kg of PAA. The hydrolysis of SP led to the formation of hydrogen peroxide, whose interaction with TAED led to the formation of PAA *in vivo*. Citric acid and ethylenediaminetetraacetic acid (EDTA) were also used to balance the pH of the water according to values normally found on farms and to stabilize the newly formed PAA. Random samples of water were collected, and pH was measured throughout the study to ensure that values were in the range between 6.6 and 7.4. Both feed and water were offered *ad libitum* to the piglets, which had free access to both feeders and drinkers throughout the animal study.

**Table 1:** Treatments used throughout the trial.

**Treatment code**	**Description**	**Active ingredient concentration**
0 ppm	Negative control	No ZnO, no PAA
ZnO	In-feed supra-nutritional ZnO (positive control)	3,100 mg/kg of in-feed ZnO
50 ppm	In-water PAA, delivered via precursors' hydrolysis	50 mg/kg of PAA (prepared mixing TAED 0.10 g/L, SP 0.2 g/L, EDTA 0.05 g/L, and citric acid 0.1 g/L)
150 ppm	In-water PAA, delivered via precursors' hydrolysis	150 mg/kg of PAA (prepared mixing TAED 0.30 g/L, SP 0.6 g/L, EDTA 0.05 g/L, and citric acid 0.3 g/L)

We inspected the pens and recorded the FS daily as a measure of the eventual diarrheal status, as detailed in Table 2. From this, the weekly fecal score average (i.e., from day 0 to day 7 and from day 7 to day 14) was used for downstream statistical analysis. Feed intake (FI) and water intake (WI) were measured daily, whereas body weight (BW) was measured at days 0, 4, 7, 11, and 14 to estimate body weight gain (BWG). FI and WI were measured by calculating the difference in weight of feed and water given to the piglets and the weight of feed and water left at the end of each 24-h period, as measured by weighing the feeder and drinker reservoirs while taring to the weight of the empty containers. The feed conversion ratio was calculated weekly through the ratio between the FI and the difference between the final and the initial total BW. Stomach, jejunum, ileum, caecum, and colon were dissected during post-mortem (day 14), from which a 0.25 g lumen aliquot was stored immediately on dry ice in a PowerBead Pro Tube of the QIAsymphony PowerFecal Pro DNA Kit (Cat. No. 938036, QIAGEN, Hilden, Germany) and further transferred to −80°C until DNA isolation. The latter was carried out at the SRUC Biomarkers Lab (Edinburgh, UK), where 4 μl of RNase A (Cat. No. 19101, QIAGEN, Hilden, Germany) were added to each sample after homogenization of the PowerBead Pro Tubes in a FastPrep-24™ 5G homogenizer (MP Biomedicals, Santa Ana, CA, USA) for 55 s at 5.5 m/s. Thereafter, DNA isolation was completed using QIAsymphony SP (Cat. No. 9001297, QIAGEN, Hilden, Germany). On trial day 14, gut content for the five gut locations was also used immediately to measure pH (accumet^®^ AE150, Fisher Scientific, Hampton, NH, USA).

**Table 2:** Fecal scoring used daily after morning inspection.

**Score**	**Description**
1	Firm stool shape
1.5	Soft but compact feces, e.g., clay-type
2	No formed stool, little spreading (“normal diarrhea”)
3	No formed stool, watery, readily spreading (“watery diarrhea”)
4	No formed stool, very watery, flecks of blood, rapidly spreading

### 2.2 Bacterial quantification

Absolute quantitative polymerase chain reaction (qPCR) was performed to quantify the bacterial concentration, and a linear plasmid nine-point standard curve was generated as previously described (Khattak et al., 2022), via targeting the V3 region of the 16S rRNA gene (314F: 5′-CCTACGGGAGGCAGCAG-3′; 518R: 5′-ATTACCGCGGCTGCTGG-3′). Copy number per qPCR reaction and output of the qPCR runs were further normalized by the average genes' copies found in bacterial cells (Stoddard et al., 2015) and by considering the concentration of extracted DNA and the amount used for the isolation (Singh et al., 2014), allowing to calculate the bacteria/g concentration. All the reactions were run in triplicate on a Mx3000 thermocycler (Agilent Technologies, Santa Clara, CA, United States) at 95°C for 3 min, thus at 95°C for 10 s and 60°C for 20 s for 40 cycles. Brilliant III Ultra-Fast SYBR Green qPCR Master Mix, 1 × (Agilent Technologies Santa Clara, CA, United States) was used to prepare the reactions in a total of 20 μl, including 1 ng of DNA template and 200 nmol/L of each primer. A triplicate negative control was added to each plate, while quantification efficiency was calculated using the standard curve slope (*m*) and taking into consideration the *R*^2^ of the linear regression used to interpolate the sample target concentration. In general, m values of −3.1 ± 0.08, *R*^2^ of 0.95 ± 0.005, and efficiency of 110.4 ± 3.98 were measured throughout the analyzed plates.

### 2.3 16S rRNA gene sequencing and bioinformatic analysis

DNA isolated from the stomach, ileum, and caecum of all the pigs was used for 16S rRNA gene sequencing. Library preparation and 16S rRNA 2 × 300 bp paired-end read MiSeq gene sequencing (Illumina, Santa Clara, CA, USA) were carried out by Omega Bioservices (Norcross, GA, USA). The V4 region of the bacterial 16S rRNA gene was targeted [F515b (Parada et al., 2016): 5′-163 TCGTCGGCAGCGTCAGATGTGTATAAGAGACAGGTGYCAGCMGCCGCGGTAA-3′; R806b (Apprill et al., 2015): 5′-GTCTCGTGGGCTCGGAGATGTGTATAAGAGACAGGGACTACNVGGGTWTCTAA-3′], and the libraries were prepared via both amplicon PCR and index PCR, the latter to incorporate adapters and barcodes. A total of two library-preparation negative controls were included in the sequencing pipeline.

Bioinformatic analysis was carried out in QIIME2 v2022.2 (Bolyen et al., 2019), where FASTQ paired-end demultiplexed reads were imported and joined via VSEARCH (Rognes et al., 2016), and quality was filtered with a minimum Phred score of 20 (McKinney, 2010; Bokulich et al., 2013). Deblur was used to denoise the reads (Amir et al., 2017), applying a trimming length of 250, thus an even sequence depth of 10,581 allowed to retain 31.03% (1,523,664) of the reads in 98.63% of the samples (i.e., all the 144 experimental samples) before carrying out diversity analysis, with richness and Shannon's diversity (Anderson, 2001; Kim et al., 2017) calculated for α-diversity and Bray–Curtis dissimilarities and Jaccard similarity index (Jaccard, 1908; Bray and Curtis, 1957) used to analyze β-diversity. The q2-feature-classifier plugin with a Naïve Bayes classifier was trained using the F515b/R806b primers and the Silva database (138, 99% of similarities) (Pruesse et al., 2007; Pedregosa et al., 2011; Bokulich et al., 2018) and was used to assign the taxonomy, while metagenome functional prediction was carried out using the full pipeline of the phylogenetic investigation of communities by reconstruction of unobserved states (PICRUSt2) software (Douglas et al., 2020), based on the marker gene (16S rRNA) alignment to the Kyoto Encyclopedia of Genes and Genomes (KEGG) database (Kanehisa et al., 2016a,b), generating a list of KEGG orthologs (Kanehisa and Goto, 2000; Kanehisa, 2019; Kanehisa et al., 2023) as output.

### 2.4 Statistical analysis

Statistical analysis was performed using R V 4.2.3 (R Core Team, 2022) in RStudio V 2023.03.0. LMM was fitted using the package “lme4” (Bates, 2010), calculating the *P*-value via type III ANOVA using Satterthwaite's method through the package “lmerTest” (Kuznetsova et al., 2017). Treatment and/or time/gut location effects on FS, performance data, pH, and α-diversity were calculated via fitting the LMM using longitudinal data, where possible, considering treatment and time as fixed effects and the hierarchy of rooms, pens, and pigs as random effects. Furthermore, we tested a total of four orthogonal contrast statements, assessing the following comparisons: 0 ppm vs. all the treatments, ZnO vs. PAA treatments, and 50 ppm vs. 150 ppm. PERMANOVA (Anderson, 2001) in QIIME 2 was used to assess the eventual differences in β-diversity, which also took into account the false discovery rate, producing both a *P*-value and a *Q*-value. QIIME2 artifacts were imported into R via the “qiime2R” package in R (Bisanz, 2018).

Differential abundance analysis was carried out using the microbiome multivariable associations with linear models (MaAsLin 2) pipeline in R (Mallick et al., 2021), where taxonomical data were normalized through the cumulative-sum scaling (CSS) method and analyzed via the compound Poisson linear model (CPLM), with treatment as a fixed effect and the room/pen/pig hierarchy as a random effect. Whereas ortholog data were normalized with the trimmed mean of M-values (TMM) method and analyzed with the zero-inflated negative binomial model (ZINB), it was reported to give the best performance with metagenomic data (Pereira et al., 2018). Both treatment (categorical) and arithmetic FS pen-mean (numerical) were used as fixed effects and the room/pen/pig hierarchy as random effects, while MaAsLin 2 also generated significance values for both *P* and *Q* values. The following describes the methodology used to identify orthologs that were either enriched or less abundant in connection to the improved phenotype. Significant (i.e., *P* < 0.05, *Q* < 0.05) ZINB negative correlations between the FS and ortholog TMM abundance would indicate an improved phenotype (i.e., lower FS) in connection to an enriched predicted ortholog. Significant positive correlations between the categorical variables (treatment) and ortholog abundance would also indicate an improved phenotype in connection to an increased ortholog abundance. In the opposite way (e.g., positive FS correlation), we identified the correlations between the orthologs with decreased abundance and the improved phenotype. Thus, we created Venn diagrams using the package “ggvenn” in R (Yan, 2023) and exported the intersection table to a .txt file via the package “gplots” in R (Warnes et al., 2022), so that we could be able to indicate which specific orthologs were either enriched or less abundant in the intersections between “FS:ZnO:50 ppm:150 ppm,” together with the intersections “ZnO:50 ppm:150 ppm,” “FS:ZnO,” “FS:50 ppm,” and “FS:150 ppm” (i.e., intersections linked to the improved PWD phenotype).

### 2.5 Power calculation for sample size determination

The FS was the most relevant variable relative to the hypothesis of this study, and it was recorded daily from 48 piglets (i.e., 12 per treatment) and analyzed as a weekly average longitudinally, with treatment and time as a fixed effect and room/pen as a random effect [fecal score ~ treatment · time + (1|room/pen)]. Therefore, the power calculation was based on the expected fecal score values based on expected observations (i.e., 1.15 ± 0.1 ≤ fecal score ≤ 1.8 ± 0.1).

A data frame was assembled in R V4.2.3 (R Core Team, 2022), containing randomly generated fecal score values; thus, the LMM was fitted using the *lmer* function of the lme4 package (Bates et al., 2015), considering treatment and time as fixed effects and the hierarchy of room/pen as random effects. The *P*-value for the LMM was calculated based on Satterthwaite's method using the package *lmerTest* (Kuznetsova et al., 2017), whereas the power was calculated based on 10,000 simulations via the package *simr* (Green and Macleod, 2016). The resulting power for the variable fecal score and the predictor treatment (95% confidence interval) was 82.62% (81.86–83.36).

## 3 Results

### 3.1 Improvement of post-weaning diarrhea and effect on pH and bacterial abundance

**Figure 1:**
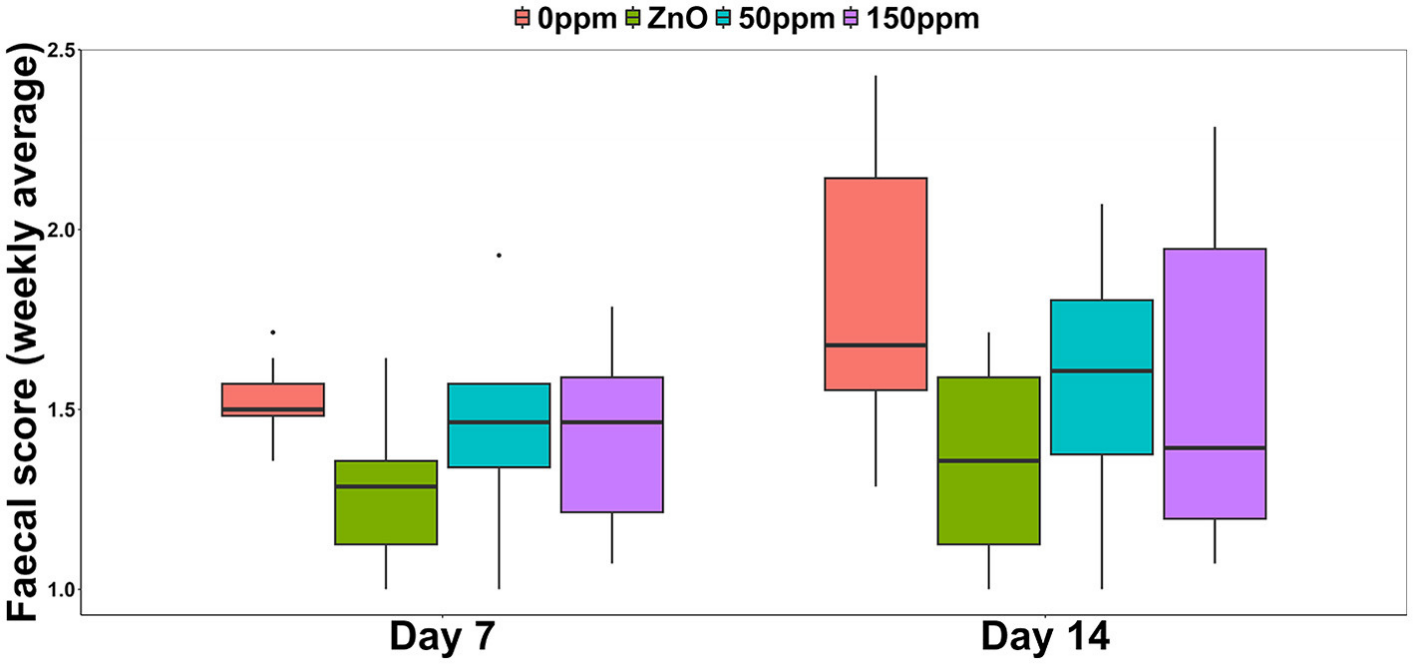
Weekly average of the fecal score among the four treatments.

Both treatment, *F*_(3, 44)_ = 4.78 (*P* < 0.05), and time, *F*_(1, 44)_ = 11.54 (*P* < 0.05), had a significant effect on the weekly average FS, with generically higher and therefore worse values observed during the second week (Figure 1). Over the first 7 days, the FS of the 0 ppm group (1.52 ± 0.1) was significantly higher than ZnO (1.26 ± 0.2, *P* < 0.05), and while both the 50 ppm (1.43 ± 0.25) and the 150 ppm (1.43 ± 0.22) groups showed a small amelioration in the diarrhetic status, this was not significantly lower than 0 ppm. At day 14, both ZnO (1.36 ± 0.26) and 50 ppm (1.57 ± 0.31) groups had better (i.e., lower) FS values than 0 ppm (1.80 ± 0.37), while FS in the 150 ppm group (1.57 ± 0.44) tended to be lower than 0 ppm (*P* = 0.05). The contrast analysis revealed that at day 7, FS tended to be lower in ZnO compared to both PAA treatments (*P* = 0.09). At day 14, the FS of the 0 ppm group was significantly higher than the rest of the treatments (*P* < 0.05), and the FS values of the ZnO group were significantly lower than those of the 50 and 150 ppm groups (*P* < 0.05).

The pH was statistically different throughout the five gut locations analyzed (i.e., stomach, jejunum, ileum, caecum, and colon), *F*_(4, 200)_ = 502.96 (*P* < 0.05), and the interaction between treatment and gut location was significant for pH, *F*_(12, 200)_ = 2.23 (*P* < 0.05). In the stomach, both 50 ppm (3.44 ± 0.70) and 150 ppm (3.62 ± 0.80) groups had significantly lower pH than 0 ppm (4.17 ± 0.50, *P* < 0.05); moreover, it was found that the stomach pH in the 0 ppm group was significantly higher than that of the rest of the treatments according to the contrast analysis (*P* < 0.05). On the other hand, the stomach pH of the ZnO group (3.88 ± 0.74) was significantly higher than that of both PAA groups, according to contrast analysis (*P* < 0.05). No further significant differences were found in the rest of the gut locations (Table 3).

**Table 3:** Average ± standard deviation of both pH and log_10_ bacterial concentration (bacteria/g) measured throughout the five gut locations analyzed and among the four treatments.

	**pH**				**Log**_**10**_ **Bac/g**			
	**0 ppm**	**ZnO**	**50 ppm**	**150 ppm**	**0 ppm**	**ZnO**	**50 ppm**	**150 ppm**
Stomach	4.17 ± 0.5^A^	3.88 ± 0.74^AB^	3.44 ± 0.7^B^	3.62 ± 0.8^B^	8.26 ± 0.44	8.37 ± 0.51	7.93 ± 0.46	8.02 ± 0.90
Jejunum	6.27 ± 0.34	6.45 ± 0.33	6.35 ± 0.37	6.34 ± 0.26	7.67 ± 1.12	8.02 ± 1.05	7.49 ± 0.90	7.52 ± 0.73
Ileum	6.96 ± 0.42	7.20 ± 0.34	7.08 ± 0.25	7.12 ± 0.25	8.34 ± 1.13	8.76 ± 0.75	8.13 ± 0.83	8.30 ± 0.84
Caecum	5.34 ± 0.35	5.60 ± 0.39	5.51 ± 0.26	5.53 ± 0.35	10.15 ± 0.85	10.44 ± 0.16	10.46 ± 0.17	10.45 ± 0.21
Colon	5.52 ± 0.33	5.66 ± 0.39	5.60 ± 0.25	5.58 ± 0.38	10.51 ± 0.11	10.45 ± 0.17	10.56 ± 0.15	10.55 ± 0.11

Log10 Bac/g, log10 bacterial concentration (bacteria/g); ZnO, zinc oxide.

Sample size for all the observations was n = 48. Different capital superscript letters in the same row for the different variables (i.e., pH or log_10_ bacteria/g) indicate statistically significant differences (P < 0.05). The table depicts significant differences between the groups only if the main P-value calculated for the model and described throughout the text was lower than the significance level of 0.05.

The bacterial concentration, hereafter reported in brackets as the average ± standard deviation of log_10_ bacteria/g, differed significantly between gut locations, *F*_(4, 176)_ = 232.99 (*P* < 0.05). Indeed, the overall concentration in the stomach (8.15 ± 0.21) and ileum (8.38 ± 0.27) was higher than that of the jejunum (7.67 ± 0.24, *P* < 0.05) and lower than that in the caecum (10.38 ± 0.15, *P* < 0.05) and in the colon (10.52 ± 0.05, *P* < 0.05). Bacterial concentration in the jejunum was significantly lower than the rest of the gut locations (*P* < 0.05); however, no statistically significant differences were found between ileum and stomach content and caecum and colon content. According to the linear mixed model (LMM), the treatment did not have an overall significant effect on the bacterial concentration throughout the five gut locations when compared to 0 ppm (Table 3).

### 3.2 Treatment-driven changes in microbial diversity

We isolated the genomic DNA from the luminal content of the stomach, ileum, and caecum of the 48 piglets at the end of the study (day 14), which we used to carry out 16S rRNA gene sequencing. A total of 20,910,326 paired-end reads were imported in QIIME2, with a range going from 91,663 to 247,788 reads per sample for the least and most represented samples, respectively. In parallel, the two negative controls (i.e., library preparation controls) accounted for 3,081 and 604 reads, respectively. A total of 11,166,017 (53%) were retained after joining and quality filtering, while a total of 3,041 features were identified after Deblur denoising, with a median frequency per sample of 34,075 and 10,581 features in the least represented sample, whereas only 134 and nine futures were found in the two negative controls, respectively.

**Figure 2:**
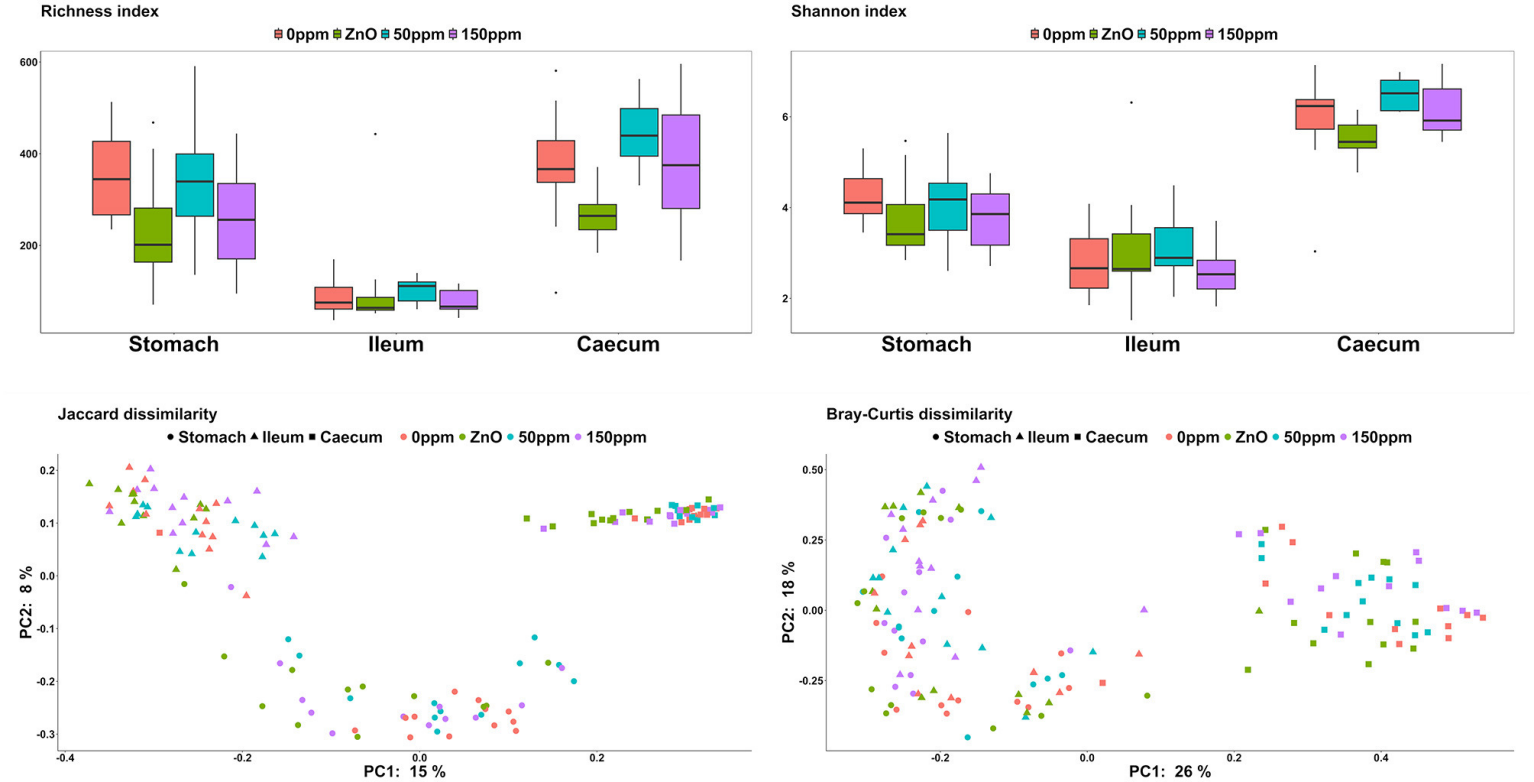
Microbial α-diversity expressed through the richness and Shannon indexes and β-diversity expressed through the Jaccard and Bray–Curtis dissimilarity indexes among the treatment groups and in the different gut locations analyzed.

Both treatment, *F*_(3, 15_) = 5.60, and gut location, *F*_(2, 112)_ = 119.16, had a significant effect on richness (*P* < 0.05, Figure 2). Furthermore, the interaction between treatment and gut location also had a significant effect on this variable, *F*_(6, 112)_ = 2.99 (*P* < 0.05). In the stomach, the ZnO group was associated with the lowest richness value (235.92 ± 124.79), which was significantly different from 0 ppm (351.25 ± 99.75, *P* < 0.05). The 150 ppm group (258.75 ± 114.54) also had a lower richness index than 0 ppm, while the latter did not differ from the 50 ppm group (334.92 ± 136.96). According to the contrast analysis, the richness for 0 ppm was significantly higher than the rest of the treatments (*P* < 0.05), whereas the richness of the ZnO group tended to be lower compared to the two PAA groups (*P* = 0.06) and 50 ppm also tended to be associated with a higher richness when compared to 150 ppm (*P* = 0.05). No significant differences were found in the ileum, where the overall richness index throughout the treatments was 92.48 ± 59.86. In the caecum, the richness associated with the ZnO group (267.5 ± 53.26) was lower than that of the 0 ppm group (373.33 ± 125.67, *P* < 0.05), while the 50 ppm group tended toward higher values (445.5 ± 67.52, *P* = 0.06). On the other hand, the caecal richness of the 150 ppm group (384.25 ± 139.12) was not significantly different from 0 ppm, whereas the richness of the ZnO was found to be statistically lower than the PAA treatments by the contrast analysis (*P* = 0.05). Both treatment, *F*_(2, 88)_ = 275.50 (*P* < 0.05), and the interaction of treatment and gut location, *F*_(6, 88)_ = 2.41 (*P* < 0.05), had a significant effect on the Shannon index; however, no significant differences were found in the stomach or in the ileum, where the overall diversity index was 3.97 ± 0.79 and 2.89 ± 0.84, respectively. In the caecum, 50 ppm treatment (6.49 ± 0.33) was associated with a higher trend index compared to 0 ppm (5.89 ± 1.04, *P* = 0.08), while the ZnO group (5.51 ± 0.42) was found to have a significantly lower index than both PAA treatments (*P* < 0.05).

In terms of β-diversity (Figure 2), both Jaccard and Bray–Curtis indexes in the stomach of the 0 ppm group were different from the ones in the rest of the treatments (*P* < 0.05, *Q* < 0.05), and while the microbial communities in the ZnO group were different compared to both 50 and 150 ppm (*P* < 0.05, *Q* < 0.05), there were no differences between the two PAA treatments. In the ileum, according to the Jaccard index, the communities of both 150 ppm and ZnO were different from 0 ppm (*P* < 0.05, *Q* < 0.05), and 150 ppm was dissimilar to both ZnO and 50 ppm (*P* < 0.05, *Q* < 0.05). Whereas the Bray–Curtis index pointed toward differences between the 150 ppm group and both 0 ppm and ZnO (*P* < 0.05, *Q* < 0.05), 50 ppm was dissimilar to both 150 ppm and ZnO (*P* < 0.05, *Q* < 0.05). In the caecum, both Jaccard and Bray–Curtis distance matrixes indicated that the communities in the 0 ppm group were different from the rest of the treatments (*P* < 0.05, *Q* < 0.05), and although both 50 and 150 ppm microbial communities were different from ZnO (*P* < 0.05, *Q* < 0.05), no differences were found between the two PAA treatments.

### 3.3 Compositional taxonomical differences among the different phenotypes

#### 3.3.1 Stomach

**Figure 3:**
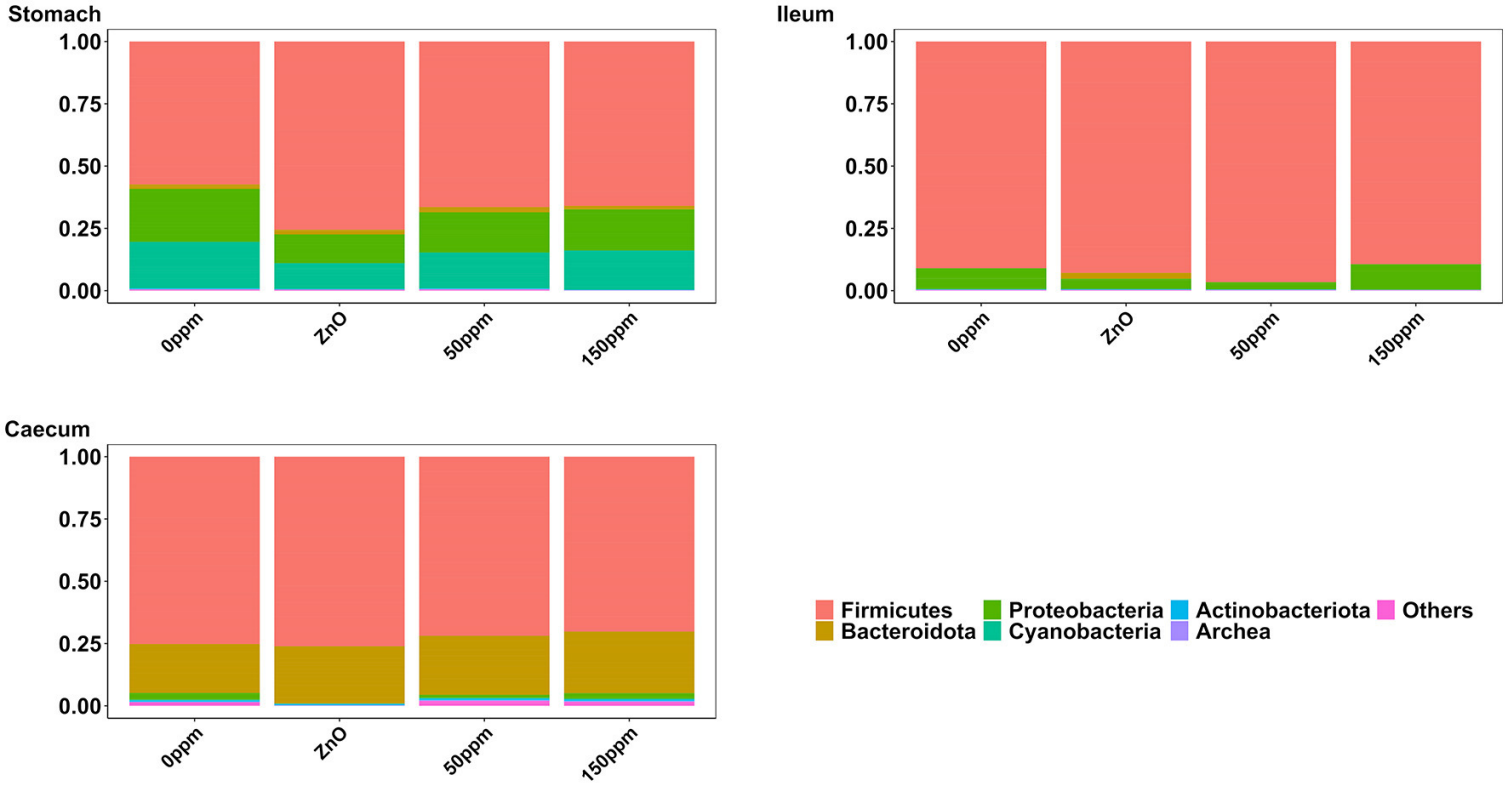
Treatment-wise phylum-level composition through the three gut locations analyzed.

**Figure 4:**
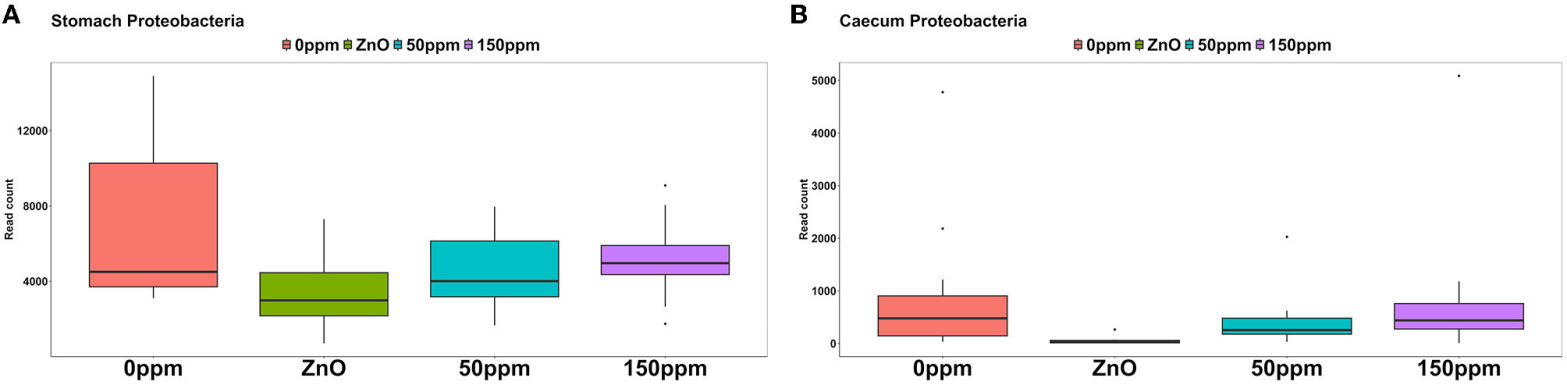
Differential abundance of Proteobacteria in the stomach **(A)** and in the caecum **(B)** throughout the treatment groups.

The most abundant phylum in the stomach was Firmicutes (Figure 3), with a relative abundance throughout the treatments of 77% ± 0.18%. Proteobacteria, the second most abundant phylum, was significantly lower in ZnO (11.54% ± 7.15%) compared to 0 ppm (21.27% ± 10.96%, *P* < 0.05, *Q* < 0.05), while it was reduced in both 50 ppm (16.15% ± 9.92%) and 150 ppm (16.5% ± 7.92%), albeit not significantly (Figure 4A). Following, Cyanobacteria (14.75% ± 8.27%), Bacteroidota (1.82% ± 1.88%), Actinobacteriota (0.34% ± 0.24%), and Archaea (0.08% ± 0.21%) were, in decrescent order, the most represented phyla throughout the treatments, with Cyanobacteria and Archaea significantly more abundant in 0 ppm (18.62% ± 8.1% and 0.08% ± 0.06%) compared to ZnO (10.31% ± 7.05% and 0.01% ± 0.02%, *P* < 0.05, *Q* < 0.05). Among the less represented phyla, Campylobacterota was less abundant in 150 ppm (0.02% ± 0.05%) compared to 0 ppm (0.21% ± 0.42%, *P* < 0.05, *Q* = 0.11), while also non-significantly reduced in 50 ppm (0.1% ± 0.13%), but not in ZnO (0.24% ± 0.43%). Moreover, Spirochaetota was significantly reduced in ZnO (0.01% ± 0.01%) when compared to 0 ppm (0.03% ± 0.03%, *P* < 0.05, *Q* < 0.05).

The genus-level compositional differences shown hereafter refer to the five most abundant genera throughout the gut locations analyzed and the treatment groups. A complete feature table, both at the phylum level and at the genus level, is provided separately (see Supplementary material 1).

**Figure 5:**
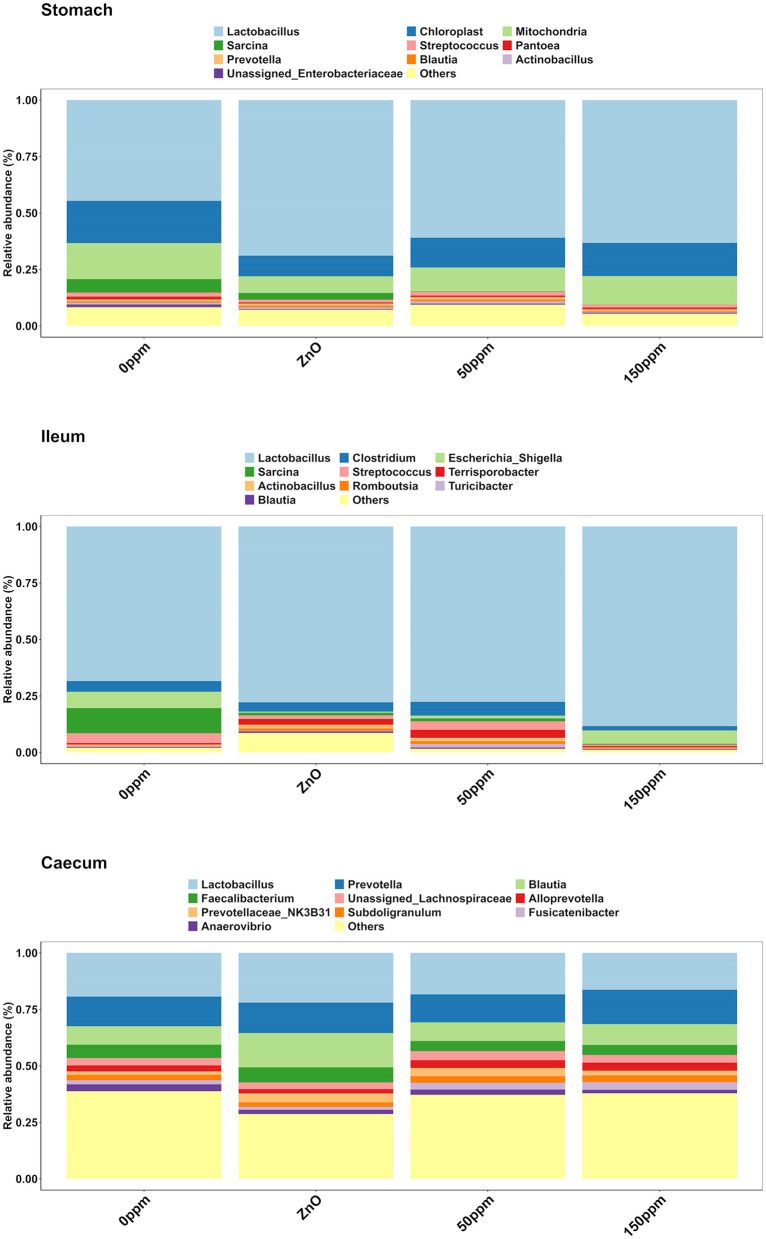
Treatment-wise genus-level composition through the three gut locations analyzed.

**Figure 6:**
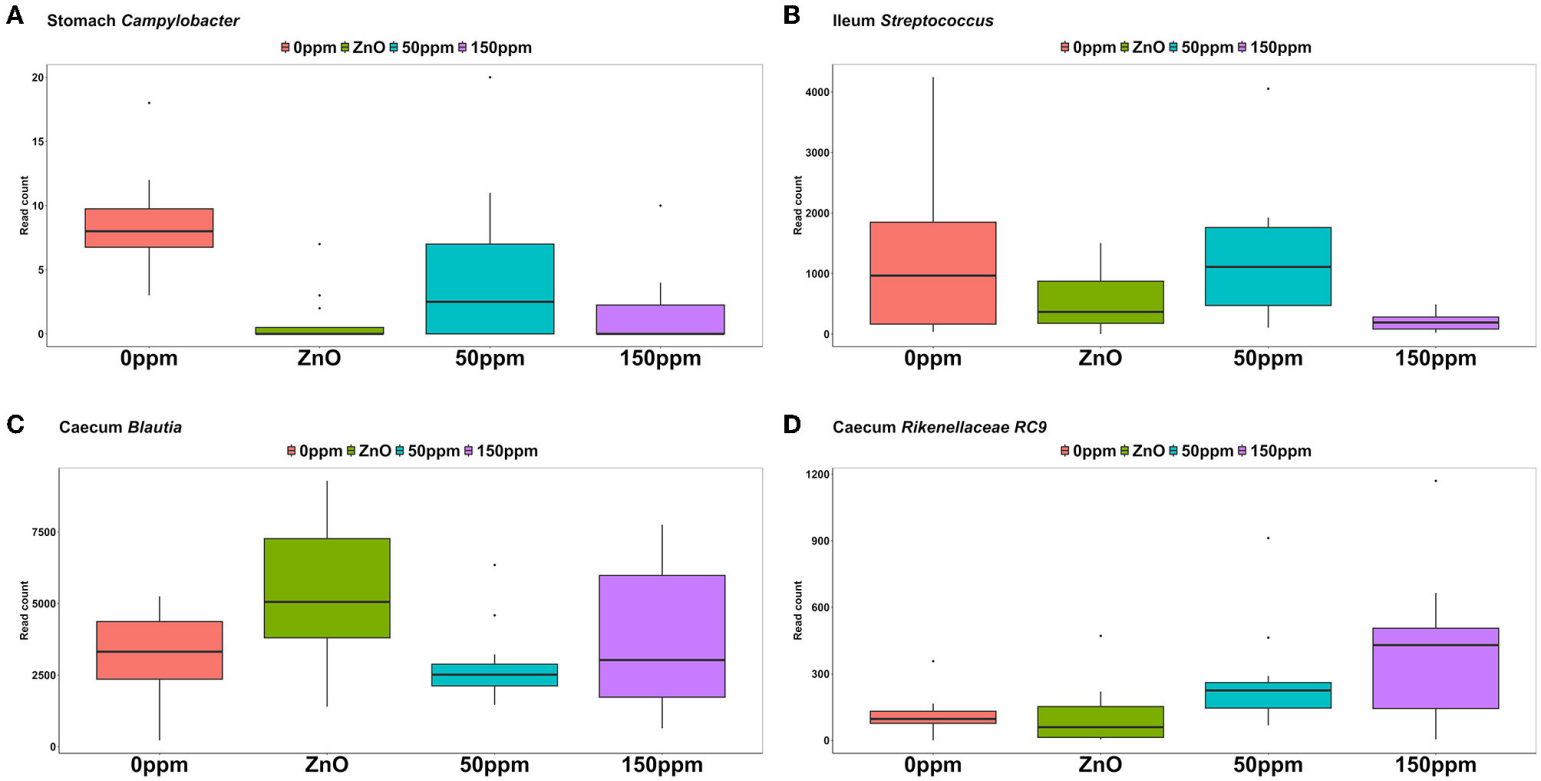
Differential abundance of *Campylobacter* in the stomach **(A)**, *Streptococcus* in the ileum **(B)**, *Blautia* in the caecum **(C)**, and *RC9* in the caecum **(D)** throughout the treatment groups.

As depicted in Figure 5, *Lactobacillus* was the most abundant genus in the four treatments, with abundance of 45.29% ± 24.82% in 0 ppm, 67.32% ± 25.92% in ZnO, 57.86% ± 21.9% in 50 ppm, and 61.05% ± 18.59% in 150 ppm, which was followed by *Chloroplast, Mitochondria, Sarcina*, and *Streptococcus*, whose general abundance among the four treatments was 14.74% ± 8.27%, 12.11% ± 6.85%, 2.00% ± 6.20%, and 1.48% ± 1.26%, respectively. *Campylobacter* was significantly reduced in both ZnO (0.004% ± 0.01%) and 150 ppm (0.01% ± 0.01%) compared to 0 ppm (0.03% ± 0.01%, *P* < 0.05, *Q* < 0.05), while being somewhat less abundant in 50 ppm (0.02% ± 0.02%, Figure 6A). Moreover, *Moraxella* and *Porphyromonas* were significantly reduced in 150 ppm compared to 0 ppm (*P* < 0.05, *Q* < 0.05), *Oscillospiraceae* UCG.002 were reduced in both ZnO (*P* < 0.05, *Q* < 0.05) and 150 ppm (*P* < 0.05, *Q* = 0.09), and *Oscillospiraceae* UCG.005 was reduced in ZnO (*P* < 0.05, *Q* < 0.05).

#### 3.3.2 Ileum

In the ileum, no significant differences were found at phylum level across the treatments, where the most abundant phylum was Firmicutes (92.44% ± 16.23%), followed by Proteobacteria (6.40% ± 15.83%), Bacteroidota (0.50% ± 3.93%), Actinobacteriota (0.17% ± 0.22%), Cyanobacteria (0.11% ± 0.14%), and Archea (0.06% ± 0.12%, Figure 3).

At the genus level (Figure 5), *Lactobacillus* was also the most abundant in the ileum, followed by *Escherichia, Clostridium*, and *Sarcina*, with abundance across the four treatments of 76.04% ± 27.41%, 4.83% ± 14.67%, 4.55% ± 9.01%, and 3.06% ± 10.82%, respectively. *Streptococcus* was significantly reduced in 150 ppm (0.62% ± 0.55%) compared to 0 ppm (4.81% ± 5.25%, *P* < 0.05, *Q* = 0.06), whereas it was somewhat less abundant in both ZnO (1.6% ± 1.3%) and 50 ppm (3.82% ± 3.27%, Figure 6B).

#### 3.3.3 Caecum

As shown in Figure 3, Firmicutes was also the most abundant phylum in the caecum, with no significant differences between treatments (73.33% ± 7.56%), followed by Bacteroidota (22.74% ± 6.06%) and Proteobacteria, which was significantly lower in ZnO (0.13% ± 0.14%) compared to 0 ppm (2.7% ± 3.83%, *P* < 0.05, *Q* < 0.05), while no differences were found when comparing its levels to the 50 ppm (1.22% ± 1.48%) and 150 ppm groups (2.23% ± 3.98%, Figure 4B). Actinobacteriota was the fourth most abundant phylum in the caecum (0.64% ± 0.36%) with no differences across treatments, followed by Cyanobacteria, significantly decreased in ZnO (0.05% ± 0.08%) compared to 0 ppm (0.37% ± 0.3%, *P* < 0.05, *Q* < 0.05) and unchanged in 50 ppm (0.31% ± 0.24%) and 150 ppm (0.37% ± 0.47%), and Archea, also decreased in ZnO (0.02% ± 0.02%) compared to 0 ppm (0.09% ± 0.09%, *P* < 0.05, *Q* = 0.10), whose abundance was non-significantly increased in 50 ppm (0.32% ± 0.3%) and 150 ppm (0.28% ± 0.36%).

Although reduced compared to the other two locations (Figure 5), *Lactobacillus* was also the most abundant genus in the caecum (18.89% ± 9.72%), followed by *Prevotella* (13.80% ± 5.48%), and *Blautia*, whose abundance was greater in ZnO (15.99% ± 8.09%) compared to 0 ppm (8.01% ± 3.9%, *P* < 0.05, *Q* < 0.05), but not in 50 ppm (8.2% ± 3.33%) or 150 ppm (9.08% ± 5.2%, Figure 6C). *Faecalibacterium* was the fourth most abundant genus in the caecum, followed by unassigned *Lachnospiraceae*, with overall abundance of 5.16% ± 3.02% and 3.38% ± 2.46%, respectively. A number of bacterial genera were differentially abundant among treatments in the caecum; however, only those whose relative abundance in the 0 ppm group was ≥0.1% were reported thereafter.

Compared to 0 ppm (0.26% ± 0.7%), *Actinobacillus* was significantly reduced to 150 ppm (0.003% ± 0.01%, *P* < 0.05, *Q* < 0.05), and ZnO (0.01% ± 0.03%, *P* < 0.05, *Q* = 0.09). *Campylobacter* was reduced in ZnO (0% ± 0%) compared to 0 ppm (0.92% ± 2.5%, *P* < 0.05, *Q* < 0.05), but the latter was not different from 50 ppm (0.57% ± 1.1%) and 150 ppm (0.53% ± 1.07%). *Anaerovibrio* was reduced in ZnO (1.49% ± 2.99%) compared to 0 ppm (2.89% ± 3.02%, *P* < 0.05, *Q* < 0.05), but not in the 50 ppm (2.25% ± 1.91%) or 150 ppm (1.57% ± 1.16%) groups.

*Clostridia* UCG.014, *Colidextribacter, Oscillospira, Intestinibacter, Ruminococcaceae* (incertae sedis), *Prevotellaceae* (NK3B31), and *Dorea* were more abundant in the ZnO group compared to 0 ppm (*P* < 0.05, *Q* < 0.05), whereas *Eubacterium* (*ruminantium* group), *Lachnospiraceae* (UCG.008), *Oribacterium, Lachnospira, Succinivibrio, Ruminococcus*, and *Gastranaerophilales* were less abundant in this group compared to 0 ppm (*P* < 0.05, *Q* < 0.05). *Rikenellaceae* RC9 was more abundant in the 150 ppm group compared to the 0 ppm group (*P* < 0.05, *Q* < 0.05, Figure 6D), whereas *Streptococcus* was reduced in the 150 ppm group compared to the 0 ppm group (*P* < 0.05, *Q* < 0.05). Finally, *Prevotellaceae* (UCG.003) was increased by 50 ppm compared to 0 ppm (*P* < 0.05, *Q* < 0.05).

Within the Archaea, *Methanobrevibacter* was increased in both 50 ppm (*P* < 0.05, *Q* < 0.05) and 150 ppm (*P* < 0.05, *Q* = 0.07) compared to 0 ppm, whereas *Methanosphaera* was reduced in ZnO (*P* < 0.05, *Q* = 0.09) compared to 0 ppm.

### 3.4 Diversity of the predicted functional metagenome throughout the treatment groups

Both treatment *F*_(3, 20)_ = 7.19 (*P* < 0.05) and gut location *F*_(2, 88)_ = 199.11 (*P* < 0.05) had a significant effect on the richness index of the predicted KEGG ortholog (KO) metagenome, which was greater in the stomach compared to both the ileum and caecum (*P* < 0.05); however, the richness did not statistically differ between the ileum and caecum. In the stomach, the predicted KO richness index accounted for 6,067.58 ± 160.4 features in the 0 ppm group, which were statistically greater than ZnO (5,601.58 ± 479.26 features, *P* < 0.05) but not than 50 ppm (5,968.83 ± 240.48 features) and 150 ppm (5,901 ± 317.19 features). The contrast analysis revealed that the predicted KO richness associated with 0 ppm tended to be greater than all the rest of the treatments (*P* = 0.07), while ZnO was significantly lower than both 50 and 150 ppm (*P* < 0.05). In the ileum, the richness in the 0 ppm group (4,675.67 ± 624.53 features) tended to be higher than ZnO (4,360.83 ± 536.66 features, *P* = 0.06), but it remained statistically unchanged when compared to 50 ppm (4,938.58 ± 193.48 features) and 150 ppm (4,492 ± 481.7 features), whereas contrast analysis confirmed that ZnO had a significant impact on KO richness when compared to both 50 and 150 ppm (*P* < 0.05), but also that the index was different when comparing 50 and 150 ppm. In the caecum, once again the KO richness of the 0 ppm group (4,895.75 ± 294.56 features) was significantly greater than the ZnO (4,297.75 ± 322.26 features), but statistically unchanged when compared to 50 ppm (4,782.17 ± 359.95 features) and 150 ppm (4,797.67 ± 289.09 features), which was also underlined by the contrast analysis showing that the index of the 0 ppm group was statistically different from the rest of the treatments (*P* < 0.05) and that the KO richness in the ZnO group was different from both 50 and 150 ppm (*P* < 0.05).

Only gut location had a statistically significant effect on the Shannon index, *F*_(2, 88)_ = 30.10 (*P* < 0.05), which was significantly lower in the ileum compared to both the stomach and caecum (*P* < 0.05). Even though treatment did not have a significant generic effect on the Shannon index, individual comparisons showed that the Shannon index in the stomach of the 0 ppm group (10.79 ± 0.27) was greater than the ZnO group (10.46 ± 0.4) but unchanged when compared to 50 ppm (10.62 ± 0.31) and 150 ppm (10.57 ± 0.28), with the contrast analysis showing that the 0 ppm group had a tendency to be associated with greater diversity than the rest of the treatments (*P* = 0.05). The Shannon index did not significantly change in the ileum, where 0 ppm (10.3 ± 0.61) was similar to ZnO (10.16 ± 0.42), 50 ppm (10.21 ± 0.31), and 150 ppm (10.22 ± 0.54). A similar situation was found in the caecum, where there were no significant changes between treatments, with the Shannon index found to be 10.66 ± 0.15 in the 0 ppm group, 10.53 ± 0.05 in ZnO, 10.62 ± 0.04 in 50 ppm, and 10.64 ± 0.14 in the 150 ppm group.

In terms of β-diversity, the predicted KO metagenome in the stomach was found to be different when compared to the 0 ppm and the ZnO groups (*P* < 0.05, *Q* < 0.05) both according to the Bray–Curtis and the Jaccard index, whereas according to the latter, 0 ppm was also different from 150 ppm (*P* < 0.05, *Q* < 0.05), whereas the predicted KO pathways in the ZnO were different from 50 ppm (*P* < 0.05, *Q* < 0.05) and tended to be different from 150 ppm (*P* = 0.05, *Q* = 0.06). In the ileum, there were no differences in β-diversity of the predicted KO metagenome between any of the treatments, according to the Bray–Curtis dissimilarity index. However, the Jaccard distance analysis showed that the KO metagenome of the ZnO group was significantly different from 50 and 150 ppm (*P* < 0.05, *Q* < 0.05) and tended to be different from 0 ppm (*P* = 0.06, *Q* = 0.07); moreover, significant differences in the KO metagenome were also found between 50 and 150 ppm (*P* < 0.05, *Q* < 0.05). The caecal KO metagenome in ZnO was different from 0 and 50 ppm, both according to the Jaccard and the Bray–Curtis indexes, while it was different from 150 ppm according to the Jaccard index (*P* < 0.05, *Q* < 0.05) and showed a trend toward difference according to the Bray–Curtis index (*P* < 0.05, *Q* = 0.052). Moreover, the KO metagenome of 50 and 150 ppm was also divergent, but only according to the Jaccard index (*P* < 0.05, *Q* < 0.05).

### 3.5 Functional correlations between the treatment-driven improved diarrhetic phenotype and the Kegg orthology metagenome

**Figure 7:**
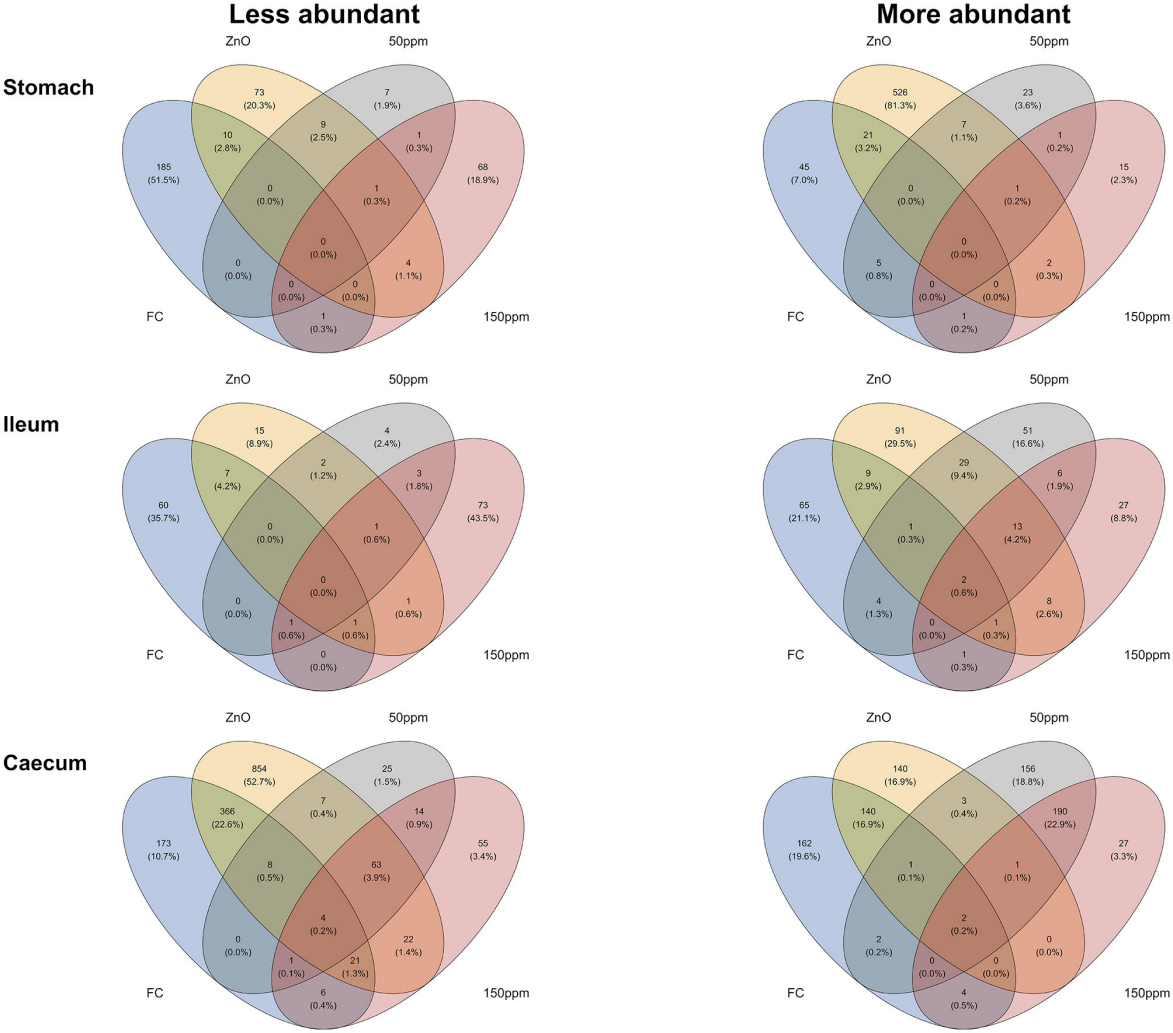
Venn diagrams depicting the intersections between the different phenotypes observed and the predicted KO orthologs.

As detailed in the Methods, Venn diagram intersections were used to unravel the correlations between differentially abundant orthologs and improved PWD phenotypes (Figure 7). Therefore, the sections hereafter describe the predicted orthologs found to be more or less abundant in connection to the improved phenotype through the different gut locations analyzed. A complete list of the orthologs found to be enriched or less abundant through the described intersections can be found in Supplementary materials 2, 3.

#### 3.5.1 Stomach

In the stomach, the predicted ortholog K03382 (hydroxydechloroatrazine ethylaminohydrolase) was found to be significantly enriched in all treatments compared to 0 ppm, whereas K10954 (zona occludens toxins) was significantly decreased in all treatments compared to 0 ppm. A total of 21 predicted orthologs were found to be significantly more abundant, both with better FS and when comparing ZnO to 0 ppm. These predicted orthologs encoded for enzymes (K18029 nicA, K00527 rtpR, K18916 ptxD, K08483 ptsI, K01187 malZ, K01258 pepT, K08659 pepDA, K03152 thiJ, K01502 E3.5.5.7, K01644 citE, and K11788 ADE5), transporters (K15770 cycB, K17074 lysX2, K17076 lysY, K08483 ptsI, K02761 celB, K02796 manZ, K06199 crcB, K03315 nhaC), peptidases, and inhibitors (K07010 putative glutamine amidotransferase, K03152 thiJ, K08659 pepDA, and K01258 pepT). On the other end, 10 predicted orthologs were less abundant when analyzing the same intersection, seven of which encoded for enzymes (K0002, K00094, K00518, K00624, K03896, K03278, K10216), while K18918 (antitoxin RelB) encoded for prokaryotic defense system/transcription factors, K03278 (waaI) was involved in lipopolysaccharide biosynthesis, and K14683 (SLC34A) encoded for transporter/exosome. A total of five predicted orthologs were significantly more abundant, both when comparing the 50 ppm group to the 0 ppm group and in pigs with better FS. Of these, K19714 (kdnB) encoded for an enzyme also involved in lipopolysaccharide biosynthesis, K08713 lctB encoded for a potassium channel transporter, K14087 echB (ech hydrogenase subunit B), and both small acid-soluble spore proteins K and L (K06428 sspK and K06429 sspL) were not assigned to any hierarchical class. Finally, one predicted ortholog (K08299 caiD; crotonobetainyl-CoA hydratase) was significantly more abundant, and one predicted ortholog (K19052 hepC; heparan-sulfate lyase) was significantly less abundant in pigs administered 150 ppm compared to 0 ppm and with improved FS.

#### 3.5.2 Ileum

In the ileum, two predicted orthologs encoding for enzymes (K00517; CYP81F and K14665; amhX) were significantly more abundant both in pigs with better FS and in all the treatment groups when compared with 0 ppm.

Whereas, a total of 13 predicted orthologs were significantly more abundant in the ZnO, 50 ppm, and 150 ppm groups compared to 0 ppm, among which were K19224, encoding for peptidoglycan biosynthesis and degradation protein lytE; K03200, encoding for the secretion system protein virB5; K00685 encoding for the arginyl-tRNA–protein transferase ATE1; K19079 encoding for the cationic antimicrobial peptide transport system ATP-binding protein vraF; K17243 encoding for the transporter proteins aguG; and K07244 encoding for mgtE-like transporter; and six predicted orthologs encoding for enzymes (K00038 E1.1.1.53; K03333 choD; K00354 E1.6.99.1; K13942 hmd; K00918 pfkC; and K00333 nuoD). On the other hand, only one predicted ortholog (K01210 E3.2.1.58) was significantly less abundant in the same intersection, involved in the starch and sucrose metabolism.

A total of nine predicted orthologs were significantly more abundant in the ZnO group when compared to 0 ppm, among which K06294 and K06313 encoding for the spore germination proteins gerD and ypeB, and orthologs encoding for the transporters fluoroquinolone transport system permease protein, yqgE, oprO_P and dmsC, while seven predicted orthologs were significantly less abundant in the same intersection, such as K16850; uxaA2 (altronate dehydratase large subunit), K18210; tfrB (fumarate reductase (CoM/CoB) subunit B), K00202; fwdC (formylmethanofuran dehydrogenase subunit C), K17830; GGR (digeranylgeranylglycerophospholipid reductase), K07151; STT3 (dolichyl-diphosphooligosaccharide–protein glycosyltransferase), K03231; EEF1A (elongation factor 1-α) and the uncharacterized ortholog K09003.

Only four predicted orthologs were significantly more abundant in the 50 ppm group compared to the 0 ppm group, which was also associated with good FS values, and these were encoded for the transporter mdeA (K18936) and the enzymes crtP, TPMT, and crtQ (K10210, K00569, and K10211).

On the other hand, K01727; hysA (hyaluronate lyase) was significantly more abundant in the 150 ppm group compared with 0 ppm and with pigs with better FS.

#### 3.5.3 Caecum

The predicted orthologs K01134, encoding for arylsulfatase A (ARSA), and K09799 (uncharacterized) were enriched in pigs with better FS and in all the treatments compared to 0 ppm, while two predicted orthologs (K09744 and K09966) encoding for uncharacterized proteins, K01167, encoding for the ribonuclease T1 (RNASA), and K02657, encoding for the twitching motility two-component system response regulator (pilG), were less abundant in the same intersection.

While only the predicted ortholog K06434, encoding for the small acid-soluble spore protein, thioredoxin-like protein (tlp), was significantly more abundant in pigs in the ZnO, 50 ppm, and 150 ppm groups when compared to 0 ppm, a total of 63 predicted orthologs were significantly less abundant within the same intersection. Among these, 37 encoded for enzymes (e.g., K16652 dprE2; decaprenylphospho-β-d-erythro-pentofuranosid-2-ulose 2-reductase), nine encoded for proteins in the two-component system (e.g., K07653 mprB; two-component system, OmpR family, sensor histidine kinase MprB), eight encoded for glycosyltransferases (e.g., K14337 mptA; α-1,6-mannosyltransferase), five encoded for protein kinases (e.g., K14949 pknG; serine/threonine-protein kinase PknG), three encoded for bacterial motility proteins (e.g., K06602 flaF; flagellar biosynthesis activator protein FlaF), two encoded for transcription factors (K18136 acrR; TetR/AcrR family transcriptional regulator, multidrug resistance operon repressor), two encoded for lipid biosynthesis proteins (K12437 and K12428) and one encoded for lipopolysaccharide biosynthesis protein (K13013 wbqV; O-antigen biosynthesis protein).

The FS:ZnO caecal intersection was associated with the majority of the predicted significant orthologs compared to the rest of the intersections and the rest of the gut locations. In total, 140 and 366 predicted orthologs were found to be significantly increased or decreased in association with this intersection, respectively. The 140 enriched orthologs were found to encode for 84 pathways or 22 functional hierarchies, among which three antimicrobial resistance genes (K02172 blaR1; bla regulator protein, K18345 vanSB; two-component system, OmpR family, sensor histidine kinase VanS and K18344 vanRB; two-component system, OmpR family, response regulator VanR), the bacterial motility proteins pilT (K02669) and pilD (K02654), the lipid biosynthesis protein (K00645; fabD), and peptidoglycan biosynthesis and degradation proteins (K01448; amiABC). On the other hand, the 366 orthologs significantly reduced within this intersection encoded for proteins involved in a total of 87 pathways or 28 functional hierarchies, among which 16 bacterial motility proteins (e.g., K02411 fliH; flagellar assembly protein FliH), 16 proteins involved in secretion systems (e.g., K03195 virB10; type IV secretion system protein VirB10), eight peptidoglycan biosynthesis and degradation proteins (e.g., K06078 lpp; murein lipoprotein), two proteins involved in prokaryotic defense systems (e.g., K09159 cptB; antitoxin CptB), and two antimicrobial resistance genes (e.g., K19212 blaOXA-63; β-lactamase class D OXA-63).

Only two predicted orthologs were found to be significantly increased in pigs in the 50 ppm range and associated with improved FS; one of them (K00210) was unassigned, and the other (K01966 PCCB) is propionyl-CoA carboxylase beta chain. On the other hand, four predicted orthologs were significantly more abundant within the FS: 150 ppm intersection, such as K16147 glgE; starch synthase, maltosyl-transferring, K00500 phhA; phenylalanine-4-hydroxylase, K12908 phpF; phosphonoformate cytidylyltransferase, and K00737 MGAT3; β-1,4-mannosyl-glycoprotein β-1,4-N-acetylglucosaminyltransferase, while six predicted orthologs were significantly less abundant within the same intersection [K06044 treY; (1->4)-α-d-glucan 1-α-d-glucosylmutase, K01236 treZ; maltooligosyltrehalose trehalohydrolase, K13923 pduL; phosphate propanoyltransferase, K03343 puo; putrescine oxidase, K01561 dehH; haloacetate dehalogenase, and K06985 aspartyl protease family protein].

### 3.6 Interventions' effects on performance

Table 4 summarizes the performance variables measured throughout the experiment. Time, *F*_(1, 42.9)_ = 98.3 (*P* < 0.001), had a significant effect on log_10_ transformed g/day weekly body weight gain (BWG); similarly, only time had a significant effect on log_10_ transformed g/day feed intake, *F*_(1, 35)_ = 93.3 (*P* < 0.001). No statistically significant differences were found between the three treatments and the control during the second week, both in terms of BWG and FI.

**Table 4:** Summary of the different performance parameters.

	**Treatment**			
	**0 ppm**	**ZnO**	**50 ppm**	**150 ppm**
**Body weight gain (g/day)**				
Days 0–7 (*n* = 48)	274.05 ± 108.6	123.93 ± 170.47	177.26 ± 141.33	202.62 ± 123.35
Days 7–14 (*n* = 48)	516.9 ± 120.06	490.6 ± 140.3	450.83 ± 129.4	441.43 ± 118.84
**Pen-level feed intake (g/day)**				
Days 0–7 (*n* = 24)	546.17 ± 117.5	360.67 ± 175.64	406.21 ± 121.95	429.4 ± 164.18
Days 7–14 (*n* = 24)	1,037.98 ± 96.32	946.36 ± 303.6	927.6 ± 200.96	987.86 ± 181.47
**Pen-level water intake (kg)**				
Days 0–7 (*n* = 24)	12.7 ± 2.9	10.74 ± 2.2	9.8 ± 3.15	9.79 ± 2.06
Days 7–14 (*n* = 24)	20 ± 2.8	16.76 ± 3.54	16 ± 5.03	15.97 ± 4.47
**Pen-level FCR**				
Days 0–7 (*n* = 20)	1.03 ± 0.16	1.00 ± 0.09	0.99 ± 0.09	1.00 ± 0.11
Days 7–14 (*n* = 24)	1.03 ± 0.19	0.96 ± 0.09	1.04 ± 0.1	1.16 ± 0.33

In general, water intake (WI) in all treatments increased during the second week (17.81 kg ± 1.91 kg) compared to the first one (10.76 kg ± 1.37 kg), as confirmed by the statistically significant LMM time effect, *F*_(1, 20)_ = 106.1 (*P* < 0.05). The decreased water intake during weeks 1 and 2 observed for the rest of the treatments compared to 0 ppm was not statistically significant.

The FCR was rather stable throughout the animal study, with overall average values of 1.01 ± 0.02 and 1.05 ± 0.08 during the first and second weeks, respectively. LMM revealed the absence of statistically significant effects of time and treatment on FCR, while also revealing the absence of significant differences between specific treatments.

## 4 Discussion

Post-weaning diarrhea in pigs is a multifactorial disease often observed most intensively during the first couple of weeks post-weaning (Rhouma et al., 2017), and ZnO interventions have been widely described during the past 30 years as a valid antimicrobial alternative (Poulsen, 1995), leading to an improvement of the symptomatology and performance (Sales, 2013). However, heavy metal-driven environmental concerns and possible AMR associations led to a ban of ZnO in pig production by the European Union in 2022 (Ekhlas et al., 2023). In this study, we tested the possible use of in-water peracetic acid delivered via the hydrolysis of the precursors SP and TAED as an antimicrobial alternative useful during the weaning phase of piglets for the control of post-weaning diarrhea. We found that both PAA concentrations led to a decrease in pH in the stomach; however, *in vitro* tests of the delivery route presented here (data not shown) indicated that PAA formation occurred at a host-physiological pH of 4.4 (Merchant et al., 2011), which thus deviates from the mode of action of the traditionally used acidifiers (Wang et al., 2022). Therefore, the lower stomach pH in pigs in both the 50 ppm and the 150 ppm groups could be due to the microbial populations inhabiting the stomach of pigs in both PAA groups. Indeed, both the Jaccard and Bray–Curtis indexes indicated that the stomach microbiota of these pigs was different from the pigs in both the ZnO and the 0 ppm groups. Some authors found that gastric pH could play a role in the control of the PWD, as they observed higher pH values in symptomatic weaning piglets, while lowered figures could correlate with a healthier phenotype due to mechanisms such as the favorable proliferation of non-pathogenic bacteria (Heo et al., 2013). In terms of bacterial concentration, we found that both the jejunal and ileal content of pigs within the ZnO group were more densely populated than that of the rest of the PAA treatments, according to qPCR, whereas according to the richness index, no differences were found between the ZnO and PAA groups in the ileum.

According to the analysis of the performance parameters, all pigs achieved the greatest body weight gain during the second week, statistically greater than the figures from week 1. Although not statistically significant according to the generic *P-*value of the LMM, the BWG of the piglets in the 0 ppm group was higher than that of the ZnO group. This seemed to be in contrast to previous findings suggesting that an improvement in fecal score would translate into an increase in body weight (Kyriakis et al., 1999), as ZnO has been shown to improve average daily gain when compared to a negative control (Lei and Kim, 2018). It ought to be mentioned that both ZnO and PAA treatments ameliorated the fecal score during the second week, as we noticed closer BWG values between these groups and the control. Although the reason behind the better BWG in the presence of elevated FS in the 0 ppm group is not clear, it might be indicative of an adaptation period necessary for the treatment to exert its effects *in vivo*, as observed for the response to ZnO in early weaned pigs (Hill et al., 2001). In addition, greater feed intake of the high-quality ration could have resulted in a greater level of hindgut fermentation, which can result in more fluid feces without any apparent detriment. Moreover, our study was carried out in absence of any subclinical challenge, which could have otherwise likely led to penalized performance, especially on high protein rations as used here (Wellock et al., 2008), thus contributing to increasing the differences in performance between the 0ppm and the treated groups. Finally, as discussed further below, the interventions impacted on microbial community levels and composition, which could have evoked variation in immune response, which in turn can impact feed intake.

In the stomach, we found a significant reduction of Proteobacteria in the ZnO group compared to 0 ppm and Campylobacterota in the 150 ppm group compared to the 0 ppm group, followed by a significant reduction in *Campylobacter* in both 150 ppm and ZnO compared to 0 ppm. Interestingly, *Campylobacter* has been previously associated with PWD (Cremonesi et al., 2022), with a known effect of ZnO in controlling this genus while leading to symptom amelioration (Kaevska et al., 2016). In our study, stomach *Campylobacter* relative abundance was much lower than that of other genera, with ~10 features in the 0 ppm group. Thus, the significance of its role in the pathogenesis of PWD during our study might be questionable. However, it has been shown that only 800 CFU of *Campylobacter jejuni* were enough to induce diarrhea symptoms in human volunteers (Kaakoush et al., 2015), and considering the physiological similarities between humans and pigs (Sciascia et al., 2016), it could be deduced that low *Campylobacter* concentrations could still be able to lead to PWD symptoms. This could suggest that the effect of both ZnO and PAA in reducing this pathogen's concentration could have contributed to the improved fecal score observed in these groups. Moreover, *Campylobacter* was also significantly reduced in the caecal content of pigs in the ZnO group. In the stomach, the opportunistic pathogen *Moraxella* (Larsen et al., 1973) was found to be reduced in the stomach of piglets at 150 ppm, which could corroborate the multifactorial aspect of PWD and underline the importance of a broad-spectrum therapeutic approach to control the dynamics of this disease.

In terms of predicted metagenomic analysis, predicted hydroxydechloroatrazine ethylaminohydrolase (atzB) and predicted zonula occludens toxin (zot) were significantly more and less abundant, respectively, in all the treatment groups compared to the control. The xenobiotic degrader atzB has been described in *Pseudomonas* species as responsible for the degradation of the herbicide Atrazine (Boundy-Mills et al., 1997), whose use in agriculture is banned in the European Union, as in addition to being toxic, it is reported to be part of the endocrine-disrupting chemicals in pigs (Yang et al., 2020). Therefore, although likely Atrazine residuals were not present in the feed provided to the pigs during this trial, it is interesting to note the significant increment of xenobiotic degraders in both PAA and ZnO pigs. Possibly, this could signify that these treatments led to a selection of microbial species able to break down toxic elements; however, whether the predicted atzB enrichment was the cause of the effect of the treatment remains unknown. The toxin zot was first described in *Vibrio cholerae* as capable of altering the structure of intercellular tight junctions, leading to mild to moderate diarrhea in humans (Fasano et al., 1991). Interestingly, zot has also been described in *Campylobacter* species as leading to intestinal epithelial barrier damage (Liu et al., 2016). Therefore, our findings, although based on predicted functional metagenomes, could indicate that, among other factors, the improved phenotype observed in both PAA and ZnO groups could be related to a decreased predicted abundance of zot in the stomach, which could have led to better intestinal integrity, likely associated with the observed improved symptomatology.

At the ileum level, *Streptococcus* was significantly reduced in the 150 ppm group. In contrast, some authors have observed enrichment in the ileum of both *Streptococcus thermophilus* and *Lactobacillus* after ZnO administration, likely caused by the tolerance to ZnO of the extracellular structure of *S. thermophilus* (Sun et al., 2022). The healthy phenotype observed in all treatment groups concurred with 15 more abundant and 1 less abundant predicted orthologs. The latter was the glucan 1,3-β-glucosidase, an enzyme involved in the degradation of complex polysaccharides, shown in high abundance in individuals consuming wheat as a staple food (Lu et al., 2021). It could be argued that a reduction in microbial groups that produce glucan 1,3-β-glucosidase in the ileum, possibly as a consequence of the treatment, contributed to the observed reduced BWG, especially during the first week, possibly due to a reduced microbial degradation ability of some of the resources. On the other hand, a series of predicted enzymes, such as amidohydrolase (amhX), which catalyze the degradation of acetylated amino acids and contribute to amino acid biosynthesis (Kempf and Bremer, 1996), were found to be more abundant in healthy pigs (i.e., ZnO, 50 and 150 ppm groups). The predicted virulence gene type IV secretion system protein (virB5) (Shirasu et al., 1990) and peptidoglycan DL-endopeptidase (lytE) (Yamamoto et al., 2003) and cationic antimicrobial peptide transport system ATP-binding protein (vraF), which confers resistance to selected cationic antimicrobial peptides (Yang et al., 2012), were all more abundant in pigs with improved diarrheal symptoms. While it is easy to imagine that an increased abundance of cell wall hydrolases such as lytE might improve the PWD symptomatology due to induced bacteriolysis (Callewaert et al., 2011), the role of virB5 and vraF might not be directly related to the observed phenotype.

A number of microbial genera were found to be differentially abundant in the caecal content among the different treatment groups. Among these genera, *Blautia* has been identified as a potential probiotic (Liu et al., 2021) and has been previously found in pigs with improved PWD phenotype after probiotic administration (Rattigan et al., 2023), likely due to its role in inflammation modulation and bacteriocin and butyrate production (Kalyana Chakravarthy et al., 2018). We found that *Blautia* was statistically more abundant in ZnO pigs, which therefore could also contribute to the phenotype observed in this group. We also found that *Actinobacillus* was significantly reduced in both 150 ppm and ZnO groups, which is in accordance with previous research correlating its decreased relative abundance in pigs with improved PWD after dietary intervention (Wang et al., 2020). On the other hand, according to previous reports, *E. coli* is usually associated with PWD (Rhouma et al., 2017); however, we found that its abundance was statistically unchanged through the treatments in the caecum. In parallel, we observed a reduction of *E. coli* relative abundance from 0 ppm (1.13% ± 2.92%) to ZnO (0.05% ± 0.11%, *P* < 0.05, *Q* = 0.24) and to 50 ppm (0.12% ± 0.25%, *P* < 0.05, *Q* = 0.17), and a somewhat higher abundance in the 150 ppm group (1.59% ± 4.07%), although these figures were not statistically significant according to the calculated *Q-*values. On the other hand, *Ruminococcus* was significantly reduced in the caecum of ZnO pigs, which is in contrast with previous findings identifying this fiber-consuming genus (Helaszek and White, 1991; Crost et al., 2018) as part of the normal post-weaning microbiota (Choudhury et al., 2021). This could suggest that the global effects of ZnO on the distal gut communities could be less beneficial than those observed in the PAA groups.

The majority of the changes in the predicted ortholog differential abundance were observed in the caecum. Two predicted orthologs were significantly more abundant in the intersection FS:ZnO:50 ppm:150 ppm and one in the interaction ZnO:50 ppm:150 ppm. These encoded for the arylsulfatase A (ARSA), which catalyzes the hydrolysis of arylsulfate ester bonds (Stressler et al., 2016), K09799 (uncharacterized), and small acid-soluble spore protein (thioredoxin-like protein, tlp), respectively, encoding for acid-soluble spore proteins in *Bacillus subtilis* (Cabrera-Hernandez et al., 1999). A total of 67 predicted orthologs were significantly less abundant in association with improved diarrheal phenotypes in all the treatment groups. Interestingly, we found that the predicted orthologs encoding for the bacterial motility proteins flaF (flagellar biosynthesis activator protein), flbT (flagellar biosynthesis repressor protein), pilG (twitching motility two-component system response regulator), and pilJ (twitching motility protein) were significantly less abundant in association with the improved phenotype. The flaF gene, conserved in α-proteobacteria, is essential for flagellin protein synthesis and therefore motility with functions in filament assembly (Llewellyn et al., 2005), while flbT intervenes in post-transcriptional regulation of flagellin genes in *Caulobacter crescentus* in the passage from swarmer to stalked cells (Anderson and Gober, 2000). On the other hand, pilG and pilJ are part of the complex signal transduction pathway controlling twitching motility, described in *Pseudomonas aeruginosa*, involved in the colonization of host tissues (Whitchurch et al., 2004). These findings could suggest that both ZnO and PAA treatments might have induced a reduction of pathogen virulence via modulation of the microbial composition.

## 5 Conclusion

In this study, we presented, to the best of our knowledge, the first application of PAA, derived from the hydrolysis of the precursors SP and TAED, in connection with reduced PWD symptoms in weaning pigs. We demonstrated that PAA led to an improved phenotype, especially during the second week of the study, similar to ZnO but at considerably reduced concentrations.

According to our findings, the effects of both PAA and ZnO were already visible in the stomach; however, the qPCR results could suggest that the broad antimicrobial effect, especially for PAA, was mainly exerted in the upper gut. We previously demonstrated that the release of PAA from unencapsulated precursors is more active in the upper gut (i.e., crop) of chickens (Galgano et al., 2023b), whereas the encapsulation of the precursors led to the release of PAA in the small intestine of broiler birds (Galgano et al., 2023a). Therefore, future possible applications of PAA in pigs as an intervention to aid the successful rearing of piglets without recourse to antibiotic treatment could include both unencapsulated and encapsulated precursors, in order to both achieve the effects observed here and to bypass the possible poor gastric stability (Palmieri et al., 2015).

We also reported here variation in a number of microbiota compositional and predicted functional changes that could be correlated to variation in PWD symptoms, emphasizing its multi-factorial nature. Most noticeably, we found a reduction of *Campylobacter* and *Moraxella* and a reduction of the predicted zot at the gastric level, which could have contributed to the observed amelioration of the diarrheal status. Moreover, we found that at the caecum level, some of the probiotic strains, such as *Blautia*, RC9, belonging to the porcine core microbiota (Holman et al., 2017) and *Prevotellaceae* (UCG.003), a key carbohydrate degrader (Adeyemi et al., 2020), were increased in ZnO by 150 and 50 ppm, respectively, suggesting a beneficial effect via downstream microbial modulation. In addition to that, we found that the abundance of some of the predicted orthologs involved in the production of motility proteins was decreased at the caecum level. Although our functional analysis was based on predictive tools, the findings presented here could represent a further step toward identifying the microbial functional profile underlying the PWD. Previous studies have shown the results of metagenomic analysis of samples collected from PWD piglets under different conditions (Gaio et al., 2021), suggesting differences in metabolic pathway abundance between pigs undergoing post-weaning challenges with enterotoxigenic *E. coli* (Apiwatsiri et al., 2022). The present study further contributes by adding prediction-based evidence toward elucidating the microbial functional role associated with an improved PWD phenotype.

## Data availability statement

The datasets used and/or analyzed during the current study are available from the corresponding author on reasonable request. The raw sequencing data (i.e., MiSeq .fastq reads) presented in the study are deposited within the European Nucleotide Archive repository, at: https://www.ebi.ac.uk/ena, accession number PRJEB71614.

## Ethics statement

The animal study was approved by SRUC internal Ethical Committee (PIG AE 20-2021). The study was conducted in accordance with the local legislation and institutional requirements.

## Author contributions

SG: Writing – original draft, Writing – review & editing. LC: Writing – review & editing. AF: Writing – review & editing. JH: Writing – review & editing.

## Supplementary material

Supplementary material 1 Feature tables at phylum and genus levels. This file depicts the composition (post-denoise features) at both phylum and genus levels throughout all the analyzed samples.

Supplementary material 2 Enriched predicted orthologs in association with improved fecal scoring FC and in comparison to 0 ppm. This file contains a table of the predicted orthologs found to be enriched in the intersections correlated with an improved diarrhetic phenotype.

Supplementary material 3 Predicted orthologs less abundant in association with improved fecal scoring FC and in comparison to 0 ppm. This file contains a table of the predicted orthologs found to be less abundant in the intersections correlated with an improved diarrhetic phenotype.

## References

[B1] Adeyemi J. A. Peters S. O. De Donato M. Cervantes A. P. Ogunade I. M. (2020). Effects of a blend of *Saccharomyces cerevisiae*-based direct-fed microbial and fermentation products on plasma carbonyl-metabolome and fecal bacterial community of beef steers. J. Anim. Sci. Biotechnol. 11, 1–10. 10.1186/s40104-019-0419-5 32095237 PMC7025411

[B2] Ahmed S. Hansen C. Dahlkilde A. L. Herrero-Fresno A. Pedersen K. S. Nielsen J. P.. (2021). The effect of colistin treatment on the selection of colistin-resistant *Escherichia coli* in weaner pigs. Antibiotics 10:465. 10.3390/antibiotics10040465 33923889 PMC8073783

[B3] Amir A. McDonald D. Navas-Molina J. A. Kopylova E. Morton J. T. Xu Z. Z.. (2017). Deblur rapidly resolves single- nucleotide community sequence patterns. mSystems 2:e00191-16. 10.1128/mSystems.00191-16 28289731 PMC5340863

[B4] Anderson M. J. (2001). A new method for non-parametric multivariate analysis of variance. Austral Ecol. 26, 32–46. 10.1046/j.1442-9993.2001.01070.x

[B5] Anderson P. E. Gober J. W. (2000). FlbT, the post-transcriptional regulator of flagellin synthesis in *Caulobacter crescentus*, interacts with the 5' untranslated region of flagellin mRNA. Mol. Microbiol. 38, 41–52. 10.1046/j.1365-2958.2000.02108.x 11029689

[B6] Apiwatsiri P. Pupa P. Sirichokchatchawan W. Sawaswong V. Nimsamer P. Payungporn S.. (2022). Metagenomic analysis of the gut microbiota in piglets either challenged or not with enterotoxigenic *Escherichia coli* reveals beneficial effects of probiotics on microbiome composition, resistome, digestive function and oxidative stress responses. PLoS ONE 17:e0269959. 10.1371/journal.pone.0269959 35749527 PMC9231746

[B7] Apprill A. Mcnally S. Parsons R. Weber L. (2015). Minor revision to V4 region SSU rRNA 806R gene primer greatly increases detection of SAR11 bacterioplankton. Aquat. Microb. Ecol. 75, 129–137. 10.3354/ame01753

[B8] Bates D. Mächler M. Bolker B. M. Walker S. C. (2015). Fitting linear mixed-effects models using lme4. J. Statist. Softw. 67, 1–48. 10.18637/jss.v067.i01

[B9] Bates D. M. (2010). lme4: Mixed-effects Modeling with R. Available online at: http://webcom.upmf-grenoble.fr/LIP/Perso/DMuller/M2R/R_et_Mixed/documents/Bates-book.pdf (accessed October 2023).

[B10] Bisanz J. E. (2018). qiime2R: Importing QIIME2 Artifacts and Associated Data into R Sessions.

[B11] Bokulich N. A. Kaehler B. D. Rideout J. R. Dillon M. Bolyen E. Knight R.. (2018). Optimizing taxonomic classification of marker-gene amplicon sequences with QIIME 2's q2-feature-classifier plugin. Microbiome 6, 1–17. 10.1186/s40168-018-0470-z 29773078 PMC5956843

[B12] Bokulich N. A. Thorngate J. H. Richardson P. M. Mills D. A. (2013). Microbial biogeography of wine grapes is conditioned by cultivar, vintage., climate. Proc. Natl Acad. Sci. 111, E139–E148. 10.1073/pnas.1317377110 24277822 PMC3890796

[B13] Bolyen E. Rideout J. R. Dillon M. R. Bokulich N. A. Abnet C. C. Al-Ghalith G. A.. (2019). Reproducible, interactive, scalable and extensible microbiome data science using QIIME 2. Nat. Biotechnol. 37, 852–857. 10.1038/s41587-019-0209-9 31341288 PMC7015180

[B14] Bonetti A. Tugnoli B. Piva A. Grilli E. (2021). Towards zero zinc oxide: feeding strategies to manage post-weaning diarrhea in piglets. Animals 11*:*642. 10.3390/ani11030642 33670980 PMC7997240

[B15] Boundy-Mills K. L. De Souza M. L. Mandelbaum R. T. Wackett L. P. Sadowsky M. J. (1997). The atzB gene of *Pseudomonas* sp. strain ADP encodes the second enzyme of a novel atrazine degradation pathway. Appl. Environ. Microbiol. 63, 916–923. 10.1128/aem.63.3.916-923.1997 9055410 PMC168384

[B16] Bray R. J. Curtis J. T. (1957). An ordination of the upland forest communities of Southern Wisconsin. Ecol. Monogr. 27, 325–349. 10.2307/1942268

[B17] Cabrera-Hernandez A. Sanchez-Salas J-. L. Paidhungat M. Setlow P. (1999). Regulation of Four Genes Encoding Small, Acid-soluble Spore Proteins in Bacillus subtilis. Available online at: www.elsevier.com/locate/gene (accessed October 2023). 10.1016/s0378-1119(99)00124-9 10333516

[B18] Callewaert L. Walmagh M. Michiels C. W. Lavigne R. (2011). Food applications of bacterial cell wall hydrolases. Curr. Opin. Biotechnol. 22, 164–171. 10.1016/j.copbio.2010.10.012 21093250

[B19] Chen X. Xu J. Ren E. Su Y. Zhu W. (2018). Co-occurrence of early gut colonization in neonatal piglets with microbiota in the maternal and surrounding delivery environments. Anaerobe 49, 30–40. 10.1016/j.anaerobe.2017.12.002 29223548

[B20] Choudhury R. Middelkoop A. Boekhorst J. Gerrits W. J. J. Kemp B. Bolhuis J. E.. (2021). Early life feeding accelerates gut microbiome maturation and suppresses acute post-weaning stress in piglets. Environ. Microbiol. 23, 7201–7213. 10.1111/1462-2920.15791 34655283 PMC9291500

[B21] Cremonesi P. Biscarini F. Castiglioni B. Sgoifo C. A. Compiani R. Moroni P.. (2022). Gut microbiome modifications over time when removing in-feed antibiotics from the prophylaxis of post-weaning diarrhea in piglets. PLoS ONE 17:e0262199. 10.1371/journal.pone.0262199 35255081 PMC8901073

[B22] Crost E. H. Le Gall G. Laverde-Gomez J. A. Mukhopadhya I. Flint H. J. Juge N.. (2018). Mechanistic insights into the cross-feeding of *Ruminococcus gnavus* and *Ruminococcus bromii* on host and dietary carbohydrates. Front. Microbiol. 9:2558. 10.3389/fmicb.2018.02558 30455672 PMC6231298

[B23] Curcio L. Luppi A. Bonilauri P. Gherpelli Y. Pezzotti G. Pesciaroli M.. (2017). Detection of the colistin resistance gene mcr-1 in pathogenic *Escherichia coli* from pigs affected by post-weaning diarrhoea in Italy. J. Glob. Antimicrob. Resist. 10, 80–83. 10.1016/j.jgar.2017.03.014 28689922

[B24] Douglas G. M. Maffei V. J. Zaneveld J. R. Yurgel S. N. Brown J. R. Taylor C. M.. (2020). (2020). PICRUSt2 for prediction of metagenome functions. Nat. Biotechnol. 38, 685–688. 10.1038/s41587-020-0548-6 32483366 PMC7365738

[B25] Ekhlas D. Sanjuán J. M. O. Manzanilla E. G. Leonard F. C. Argüello H. Burgess C. M.. (2023). Comparison of antimicrobial resistant *Escherichia coli* isolated from Irish commercial pig farms with and without zinc oxide and antimicrobial usage. Gut Pathog. 15, 1–16. 10.1186/s13099-023-00534-3 36829209 PMC9951511

[B26] Fasano A. Baudry B. Pumplin D. W. Wasserman S. S. Tall B. D. Ketley J. M.. (1991). Vibrio cholerae produces a second enterotoxin, which affects intestinal tight junctions. Proc. Natl Acad. Sci. 88, 5242–5246. 10.1073/pnas.88.12.5242 2052603 PMC51848

[B27] Gaio D. Demaere M. Z. Anantanawat K. Eamens G. J. Liu M. Zingali T.. (2021). A large-scale metagenomic survey dataset of the post-weaning piglet gut lumen. Gigascience 10:giab039. 10.1093/gigascience/giab039 34080630 PMC8173662

[B28] Galgano S. Conway L. Dalby N. Fellows A. Houdijk J. G. M. (2023a). Encapsulated peracetic acid as a valid broad-spectrum antimicrobial alternative, leading to beneficial microbiota compositional changes and enhanced performance in broiler chickens. J. Anim. Sci. Biotechnol. 14:83. 10.1186/s40104-023-00881-w 37291646 PMC10251604

[B29] Galgano S. Conway L. Maggio F. D Farthing K. Dalby N. Fellows A.. (2023b). Precursor-derived in-water peracetic acid impacts on broiler performance, gut microbiota and antimicrobial resistance genes. Poult. Sci. 102:102368. 10.1016/j.psj.2022.102368 36566657 PMC9801209

[B30] Green P. Macleod C. J. (2016). SIMR: An R package for power analysis of generalized linear mixed models by simulation. Methods Ecol. Evol. 7, 493–498. 10.1111/2041-210X.12504

[B31] Guevarra R. B. Lee J. H. Lee S. H. Seok M. J. Kim D. W. Kang B. N.. (2019). Piglet gut microbial shifts early in life: causes and effects. J. Anim. Sci. Biotechnol. 10, 1–10. 10.1186/s40104-018-0308-3 30651985 PMC6330741

[B32] Helaszek C. T. White B. a. (1991). Cellobiose uptake and metabolism by ruminococcus-flavefaciens. Appl. Environ. Microbiol. 57, 64–68. 10.1128/aem.57.1.64-68.1991 2036021 PMC182665

[B33] Heo J. M. Opapeju F. O. Pluske J. R. Kim J. C. Hampson D. J. Nyachoti C. M.. (2013). Gastrointestinal health and function in weaned pigs: a review of feeding strategies to control post-weaning diarrhoea without using in-feed antimicrobial compounds. J. Anim. Physiol. Anim. Nutr. 97, 207–237. 10.1111/j.1439-0396.2012.01284.x 22416941

[B34] Hill G. M. Mahan D. C. Carter S. D. Cromwell G. L. Ewan R. C. Harrold R. L. (2001). Effect of Pharmacological Concentrations of Zinc Oxide with or without the Inclusion of an Antibacterial Agent on Nursery Pig Performance 1. Available online at: https://academic.oup.com/jas/article/79/4/934/4682823. 10.2527/2001.794934x 11325200

[B35] Holman D. B. Brunelle B. W. Trachsel J. Allen H. K. (2017). Meta-analysis to define a core microbiota in the swine gut. mSystems 2, 1–14. 10.1128/mSystems.00004-17 28567446 PMC5443231

[B36] Jaccard P. (1908). Nouvelles recherches sur la distribution florale. Bull. Soc. Vaud. Sci. Nat. 44, 223–270.

[B37] Jiang L. Feng C. Tao S. Li N. Zuo B. Han D.. (2019). Maternal imprinting of the neonatal microbiota colonization in intrauterine growth restricted piglets: a review. J. Anim. Sci. Biotechnol. 10, 1–8. 10.1186/s40104-019-0397-7 31737268 PMC6844051

[B38] Kaakoush N. O. Castaño-Rodríguez N. Mitchell H. M. Man S. M. (2015). Global epidemiology of Campylobacter infection. Clin. Microbiol. Rev. 28, 687–720. 10.1128/CMR.00006-15 26062576 PMC4462680

[B39] Kaevska M. Lorencova A. Videnska P. Sedlar K. Provaznik I. Trckova M.. (2016). Effect of sodium humate and zinc oxide used in prophylaxis of post-weaning diarrhoea on faecal microbiota composition in weaned piglets. Vet. Med. 61, 328–336. 10.17221/54/2016-VETMED

[B40] Kalyana Chakravarthy S. Jayasudha R. Sai Prashanthi G. Ali M. H. Sharma S. Tyagi M.. (2018). Dysbiosis in the gut bacterial microbiome of patients with uveitis, an inflammatory disease of the eye. Indian J. Microbiol. 58, 457–469. 10.1007/s12088-018-0746-9 30262956 PMC6141402

[B41] Kanehisa M. (2019). Toward understanding the origin and evolution of cellular organisms. Protein Sci. 28, 1947–1951. 10.1002/pro.3715 31441146 PMC6798127

[B42] Kanehisa M. Furumichi M. Sato Y. Kawashima M. Ishiguro-Watanabe M. (2023). KEGG for taxonomy-based analysis of pathways and genomes. Nucleic Acids Res. 51, D587–D592. 10.1093/nar/gkac963 36300620 PMC9825424

[B43] Kanehisa M. Goto S. (2000). KEGG: kyoto encyclopedia of genes and genomes. Nucleic Acids Res. 28, 27–30. 10.1093/nar/28.1.27 10592173 PMC102409

[B44] Kanehisa M. Sato Y. Kawashima M. Furumichi M. Tanabe M. (2016b). KEGG as a reference resource for gene and protein annotation. Nucleic Acids Res. 44, D457–D462. 10.1093/nar/gkv1070 26476454 PMC4702792

[B45] Kanehisa M. Sato Y. Morishima K. (2016a). BlastKOALA and GhostKOALA: KEGG tools for functional characterization of genome and metagenome sequences. J. Mol. Biol. 428, 726–731. 10.1016/j.jmb.2015.11.006 26585406

[B46] Kempf B. Bremer E. (1996). A novel amidohydrolase gene from *Bacillus subtilis* cloning: DNA-sequence analysis and map position of amhX. FEMS Microbiol. Lett. 141, 129–137. 10.1111/j.1574-6968.1996.tb08374.x 8768514

[B47] Khattak F. Galgano S. Houdijk J. (2022). Bacterial concentration and *Campylobacter* spp. quantification differ when fresh or ultra-frozen samples are analysed over time using molecular biology and culture-based methods. PLoS ONE 17, e0274682. 10.1371/journal.pone.0274682 36112572 PMC9481049

[B48] Kim B. R. Shin J. Guevarra R. B. Lee J. H. Kim D. W. Seol K. H.. (2017). Deciphering diversity indices for a better understanding of microbial communities. J. Microbiol. Biotechnol. 27, 2089–2093. 10.4014/jmb.1709.09027 29032640

[B49] Kitis M. (2004). Disinfection of wastewater with peracetic acid: a review. Environ. Int. 30, 47–55. 10.1016/S0160-4120(03)00147-8 14664864

[B50] Konstantinov S. R. Awati A. A. Williams B. A. Miller B. G. Jones P. Stokes C. R.. (2006). Post-natal development of the porcine microbiota composition and activities. Environ. Microbiol. 8, 1191–1199. 10.1111/j.1462-2920.2006.01009.x 16817927

[B51] Kuznetsova A. Brockhoff P. B. Christensen R. H. B. (2017). lmerTest package: tests in linear mixed effects models. J. Stat. Softw. 82, 1–26. 10.18637/jss.v082.i13

[B52] Kyriakis S. C. Tsiloyiannis V. K. Vlemmas J. Sarris K. Tsinas A. C. Alexopoulos C.. (1999). The effect of probiotic LSP 122 on the control of post-weaning diarrhoea syndrome of piglets. Res. Vet. Sci 67, 223–228. 10.1053/rvsc.1999.0308 10607501

[B53] Laine T. M. Lyytikäinen T. Yliaho M. Anttila M. (2008). Risk factors for post-weaning diarrhoea on piglet producing farms in Finland. Acta Vet. Scand. 50, 1–11. 10.1186/1751-0147-50-21 18564407 PMC2481246

[B54] Larsen J. L. Bille N. Nielsen N. C. (1973). Occurrence and possible role of *Moraxella* species in pigs. Acta Pathol. Microbiol. Scand. B Microbiol. Immunol. 81B, 181–186. 10.1111/j.1699-0463.1973.tb00208.x 4520376

[B55] Lei X. J. Kim I. H. (2018). Low dose of coated zinc oxide is as effective as pharmacological zinc oxide in promoting growth performance, reducing fecal scores., improving nutrient digestibility and intestinal morphology in weaned pigs. Anim. Feed Sci. Technol. 245, 117–125. 10.1016/j.anifeedsci.2018.06.011

[B56] Liu F. Lee H. Lan R. Zhang L. (2016). Zonula occludens toxins and their prophages in *Campylobacter* species. Gut Pathog. 8, 1–11. 10.1186/s13099-016-0125-1 27651834 PMC5025632

[B57] Liu X. Mao B. Gu J. Wu J. Cui S. Wang G.. (2021). Blautia—a new functional genus with potential probiotic properties? Gut Microbes 13, 1–21. 10.1080/19490976.2021.1875796 33525961 PMC7872077

[B58] Llewellyn M. Dutton R. J. Easter J. O'Donnol D. Gober J. W. (2005). The conserved flaF gene has a critical role in coupling flagellin translation and assembly in *Caulobacter crescentus*. Mol. Microbiol. 57, 1127–1142. 10.1111/j.1365-2958.2005.04745.x 16091049

[B59] Lu J. Zhang L. Zhai Q. Zhao J. Zhang H. Lee Y. K.. (2021). Chinese gut microbiota and its associations with staple food type, ethnicity., urbanization. NPJ Biofilms Microbiomes 7:71. 10.1038/s41522-021-00245-0 34489454 PMC8421333

[B60] Madec F. Bridoux N. Bounaix S. Â. Cariolet R. Duval-Iah Y. Hampson D. J.. (2000). Experimental models of porcine post-weaning colibacillosis and their relationship to post-weaning diarrhoea and digestive disorders as encountered in the ^®^eld. Vet. Microbiol. 72, 295–310. 10.1016/S0378-1135(99)00202-3 10727839

[B61] Mallick H. Rahnavard A. McIver L. J. Ma S. Zhang Y. Nguyen L. H.. (2021). Multivariable association discovery in population-scale meta-omics studies. PLoS Comput. Biol. 17:e1009442. 10.1371/journal.pcbi.1009442 34784344 PMC8714082

[B62] McKinney W. (2010). “Data structures for statistical computing in Python,” in Proceedings of the 9th Python in Science Conference, eds S. Van der Walt, and J. Millman, 51–56. Available online at: http://conference.scipy.org/proceedings/scipy2010/pdfs/proceedings.pdf (accessed October 2023).

[B63] Merchant H. A. McConnell E. L. Liu F. Ramaswamy C. Kulkarni R. P. Basit A. W.. (2011). Assessment of gastrointestinal pH, fluid and lymphoid tissue in the guinea pig, rabbit and pig, and implications for their use in drug development. Eur. J. Pharm. Sci. 42, 3–10. 10.1016/j.ejps.2010.09.019 20932902

[B64] Mickiewicz B. (2022). Trends and economic forecast of sustainable development of world production of meat and meat products. VUZF Rev. 7, 135–142. 10.38188/2534-9228.22.2.14

[B65] Ou D. Li D. Cao Y. Li X. Yin J. Qiao S.. (2007). Dietary supplementation with zinc oxide decreases expression of the stem cell factor in the small intestine of weanling pigs. J. Nutr. Biochem. 18, 820–826. 10.1016/j.jnutbio.2006.12.022 17475461

[B66] Pajarillo E. A. B. Chae J. P. Balolong M. P. Kim H. B. Kang D. K. (2014). Assessment of fecal bacterial diversity among healthy piglets during the weaning transition. J. Gen. Appl. Microbiol. 60, 140–146. 10.2323/jgam.60.140 25273987

[B67] Palmieri V. Bugli F. Papi M. Ciasca G. Maulucci G. Galgano S.. (2015). VP6-SUMO self-assembly as nanocarriers for gastrointestinal delivery. J. Nanomater 2015:378786. 10.1155/2015/378786

[B68] Parada A. E. Needham D. M. Fuhrman J. A. (2016). Every base matters: assessing small subunit rRNA primers for marine microbiomes with mock communities, time series and global field samples. Environ. Microbiol. 18, 1403–1414. 10.1111/1462-2920.13023 26271760

[B69] Pedregosa F. Varoquaux G. Gramfort A. Michel V. Thirion B. (2011). Scikit-learn: machine learning in Python. J. Mach. Learn. Res. 12, 2825–2830. Available online at: https://www.jmlr.org/papers/volume12/pedregosa11a/pedregosa11a.pdf

[B70] Pereira M. B. Wallroth M. Jonsson V. Kristiansson E. (2018). Comparison of normalization methods for the analysis of metagenomic gene abundance data. BMC Genomics 19, 1–17. 10.1186/s12864-018-4637-6 29678163 PMC5910605

[B71] Popescu A. (2020). Trends in pork market in the European Union and in its main producing countries in the period 2007-2018. Sci. Papers, Ser. Manag. Econom. Eng. Agric. Rural Dev. 20, 475–488.

[B72] Poulsen H. D. (1995). Zinc oxide for weanling piglets. Acta Agric. Scand. A Anim. Sci. 45, 159–167. 10.1080/09064709509415847

[B73] Pruesse E. Quast C. Knittel K. Fuchs B. M. Ludwig W. Peplies J.. (2007). SILVA: a comprehensive online resource for quality checked and aligned ribosomal RNA sequence data compatible with ARB. Nucleic Acids Res. 35, 7188–7196. 10.1093/nar/gkm864 17947321 PMC2175337

[B74] R Core Team (2022). R: A Language and Environment for Statistical Computing. Available online at: https://www.R-project.org/ (accessed October 2023).

[B75] Rattigan R. Lawlor P. G. Cormican P. Crespo-Piazuelo D. Cullen J. Phelan J. P.. (2023). Maternal and/or post-weaning supplementation with *Bacillus altitudinis* spores modulates the microbial composition of colostrum, digesta and faeces in pigs. Sci. Rep. 13:8900. 10.1038/s41598-023-33175-2 37264062 PMC10233552

[B76] Rhouma M. Fairbrother J. M. Beaudry F. Letellier A. (2017). (2017). Post weaning diarrhea in pigs: risk factors and non-colistin-based control strategies. Acta Vet. Scand. 59, 1–19. 10.1186/s13028-017-0299-7 28526080 PMC5437690

[B77] Rognes T. Flouri T. Nichols B. Quince C. Mahé F. (2016). VSEARCH: a versatile open source tool for metagenomics. PeerJ 4:e2584. 10.7717/peerj.2584 27781170 PMC5075697

[B78] Sales J. (2013). Effects of pharmacological concentrations of dietary zinc oxide on growth of post-weaning pigs: a meta-analysis. Biol. Trace Elem. Res. 152, 343–349. 10.1007/s12011-013-9638-3 23463368

[B79] Sciascia Q. Daş G. Metges C. C. (2016). REVIEW: The pig as a model for humans: Effects of nutritional factors on intestinal function and health. J. Anim. Sci. 94, 441–452. 10.2527/jas.2015-9788

[B80] Shirasu K. Morel P. Kado C. I. (1990). Characterization of the virB operon of an *Agrobacterium tumefaciens* Ti plasmid: nucleotide sequence and protein analysis. Mol. Microbiol. 4, 1153–1163. 10.1111/j.1365-2958.1990.tb00690.x 2233252

[B81] Singh K. M. Pandya P. R. Tripathi A. K. Patel G. R. Parnerkar S. Kothari R. K.. (2014). Study of rumen metagenome community using qPCR under different diets. Meta Gene 2, 191–199. 10.1016/j.mgene.2014.01.001 25606402 PMC4287863

[B82] Stoddard S. F. Smith B. J. Hein R. Roller B. R. K. Schmidt T. M. (2015). rrnDB: improved tools for interpreting rRNA gene abundance in bacteria and archaea and a new foundation for future development. Nucleic Acids Res. 43, 593–598. 10.1093/nar/gku1201 25414355 PMC4383981

[B83] Stressler T. Seitl I. Kuhn A. Fischer L. (2016). Detection, production., application of microbial arylsulfatases. Appl. Microbiol. Biotechnol. 100, 9053–9067. 10.1007/s00253-016-7838-4 27654655

[B84] Sun Y. Ma N. Qi Z. Han M. Ma X. (2022). Coated zinc oxide improves growth performance of weaned piglets via gut microbiota. Front. Nutr. 9:819722. 10.3389/fnut.2022.819722 35284437 PMC8916703

[B85] Wang H. Long W. Chadwick D. Zhang X. Zhang S. Piao X.. (2022). Dietary acidifiers as an alternative to antibiotics for promoting pig growth performance: a systematic review and meta-analysis. Anim. Feed Sci. Technol. 289:115320. 10.1016/j.anifeedsci.2022.115320

[B86] Wang W. Wang Y. Hao X. Duan Y. Meng Z. An X.. (2020). Dietary fermented soybean meal replacement alleviates diarrhea in weaned piglets challenged with enterotoxigenic *Escherichia coli* K88 by modulating inflammatory cytokine levels and cecal microbiota composition. BMC Vet. Res. 16:245. 10.1186/s12917-020-02466-5 32664940 PMC7362456

[B87] Warnes G. R. Bolker B. Bonebakker L. Gentleman R. Huber W. Liaw A.. (2022). gplots: Various R Programming Tools for Plotting Data.

[B88] Wellock I. J. Fortomaris P. D. Houdijk J. G. M. Kyriazakis I. (2008). Effects of dietary protein supply, weaning age and experimental enterotoxigenic *Escherichia coli* infection on newly weaned pigs: performance. Animal 2, 834–842. 10.1017/S1751731108001559 22443662

[B89] Whitchurch C. B. Leech A. J. Young M. D. Kennedy D. Sargent J. L. Bertrand J. J.. (2004). Characterization of a complex chemosensory signal transduction system which controls twitching motility in *Pseudomonas aeruginosa*. Mol. Microbiol. 52, 873–893. 10.1111/j.1365-2958.2004.04026.x 15101991

[B90] Yamamoto H. Kurosawa S. I. Sekiguchi J. (2003). Localization of the Vegetative cell wall hydrolases LytC, LytE., LytF on the *Bacillus subtilis* cell surface and stability of these enzymes to cell wall-bound or extracellular proteases. J. Bacteriol. 185, 6666–6677. 10.1128/JB.185.22.6666-6677.2003 14594841 PMC262103

[B91] Yan L. (2023). ggvenn: Draw Venn Diagram by “ggplot2.”

[B92] Yang C. Song G. Lim W. (2020). Effects of endocrine disrupting chemicals in pigs. Environ. Pollut. 263:114505. 10.1016/j.envpol.2020.114505 32268228

[B93] Yang S. J. Bayer A. S. Mishra N. N. Meehl M. Ledala N. Yeaman M. R.. (2012). The Staphylococcus aureus two-component regulatory system, grars, senses and confers resistance to selected cationic antimicrobial peptides. Infect. Immun. 80, 74–81. 10.1128/IAI.05669-11 21986630 PMC3255649

