# Supplementary material for: Impact of precursor-derived peracetic acid on post-weaning diarrhea, intestinal microbiota, and predicted microbial functional genes in weaned pigs
Source: Front Microbiol. 2024 Jan 25;15:1356538. doi: 10.3389/fmicb.2024.1356538 (PMC10850238; doi:10.3389/fmicb.2024.1356538)
Supplement: Supplementary material 2 — Enriched predicted orthologs in association with improved fecal scoring FC and in comparison to 0 ppm. This file contains a table of the predicted orthologs found to be enriched in the intersections correlated with an improved diarrhetic phenotype. [file Table_2.DOCX]

Supplementary Material 2

# Enriched predicted orthologs in association with improved fecal scoring FC and in comparison to 0ppm.

| Intersection | Stomach | Ileum | Caecum |
| --- | --- | --- | --- |
| FC:ZnO:50ppm:150ppm |  | K00517, K14665 | K09799, K01134 |
| ZnO:50ppm:150ppm | K03382 | K03200, K06434, K03333, K19224, K00038, K00354, K00918, K00333, K00685, K19079, K07244, K13942, K17243 | K06434 |
| FC:ZnO | K00527, K03152, K07010, K15770, K08483, K06199, K01258, K17074, K17076, K08659, K02796, K01187, K03293, K02761, K03315, K01644, K01646, K18029, K01502, K11788, K18916 | K00856, K15862, K00185, K07221, K16906, K06294, K06313, K06314, K08222 | K00645, K00053, K01151, K01495, K00616, K01886, K03742, K00262, K03426, K03499, K04069, K02669, K01448, K00336, K03523, K06390, K06392, K06393, K00705, K03546, K06396, K00003, K03324, K01190, K07667, K03310, K02647, K17103, K04769, K00975, K07095, K02032, K06213, K16789, K06864, K06898, K02231, K00854, K02049, K01486, K02654, K00821, K02051, K07658, K02315, K03308, K04720, K00128, K01961, K02160, K07699, K01703, K01567, K07720, K07636, K07402, K00833, K10441, K07271, K18640, K01182, K10439, K06400, K01464, K07814, K04028, K01470, K10117, K00334, K01308, K03604, K10118, K12251, K09759, K01804, K03741, K18369, K10202, K10119, K10201, K06133, K03186, K03752, K16248, K19310, K02172, K04029, K01751, K04749, K03753, K01818, K02377, K04024, K10200, K07775, K02431, K01206, K14392, K12941, K04031, K06384, K02805, K00076, K04087, K01305, K06395, K00197, K03389, K03390, K05341, K15984, K06928, K10543, K08084, K13890, K18344, K13922, K13889, K07709, K05303, K07219, K19169, K06937, K07469, K10562, K01428, K09706, K03188, K03189, K03190, K03187, K01430, K14048, K17236, K03191, K18345, K11691, K01039, K01040, K09516 |
| FC:50ppm | K14087, K19714, K08713, K06429, K06428 | K10210, K10211, K18936, K00569 | K01966, K00210 |
| FC:150ppm | K08299 | K01727 | K16147, K00500, K00737, K12908 |
