# Supplementary material for: Impact of precursor-derived peracetic acid on post-weaning diarrhea, intestinal microbiota, and predicted microbial functional genes in weaned pigs
Source: Front Microbiol. 2024 Jan 25;15:1356538. doi: 10.3389/fmicb.2024.1356538 (PMC10850238; doi:10.3389/fmicb.2024.1356538)
Supplement: Supplementary material 3 — Predicted orthologs less abundant in association with improved fecal scoring FC and in comparison to 0 ppm. This file contains a table of the predicted orthologs found to be less abundant in the intersections correlated with an improved diarrhetic phenotype. [file Table_3.DOCX]

Supplementary Material 3

# Predicted orthologs less abundant in association with improved fecal scoring FC and in comparison to 0ppm.

| Intersection | Stomach | Ileum | Caecum |
| --- | --- | --- | --- |
| FC:ZnO:50ppm:150ppm |  |  | K09966, K02657, K01167, K09744 |
| ZnO:50ppm:150ppm | K10954 | K01210 | K01616, K02229, K07653, K07669, K09146, K11387, K12428, K12437, K13686, K13693, K14136, K14337, K14339, K14743, K14949, K16146, K16237, K16647, K16648, K16649, K16652, K16653, K18851, K18917, K18136, K00220, K00410, K01432, K02164, K02305, K02448, K04719, K06601, K06602, K07050, K08691, K08714, K09826, K09883, K09948, K10126, K11443, K12448, K13013, K13583, K13587, K14447, K14448, K14449, K14980, K14981, K16871, K17662, K18649, K18661, K19054, K00689, K13598, K13599, K09929, K02660, K07395, K02225 |
| FC:ZnO | K00518 K00624 K14683 K10216 K03896 K00021 K07123 K18918 K00094 K03278 | K07151, K00202, K03231, K17830, K16850, K18210, K09003 | K08086, K00249, K09771, K00363, K03776, K03717, K03809, K02170, K09921, K03598, K02498, K01584, K12262, K03656, K15539, K11719, K00313, K07275, K07122, K08310, K17247, K03690, K03597, K13634, K06077, K02402, K02403, K02423, K03586, K02441, K03576, K11472, K00163, K18850, K01638, K03747, K00855, K03756, K00344, K14682, K06186, K09862, K02199, K07115, K01682, K01069, K03749, K08484, K07147, K02686, K05501, K09801, K11074, K11075, K11076, K12152, K14055, K03087, K04044, K06219, K01659, K07740, K11750, K03600, K03806, K07320, K10763, K14393, K13288, K00112, K00113, K00631, K07262, K03554, K04765, K02453, K06999, K00982, K07278, K08305, K00285, K01920, K07708, K10109, K03831, K10108, K18778, K05541, K13283, K11900, K11901, K09791, K08304, K07153, K03683, K04691, K04774, K05365, K00998, K01716, K02521, K02560, K03314, K03573, K03607, K04770, K05803, K05851, K06212, K06918, K07261, K07479, K07773, K09891, K09893, K09897, K09899, K19227, K19228, K19229, K19230, K09902, K05590, K06957, K07648, K07751, K08312, K07638, K03578, K06078, K06203, K03577, K03732, K09857, K02024, K00362, K10111, K12058, K03634, K10680, K03611, K02455, K09937, K00795, K15257, K11903, K05802, K13821, K06181, K12339, K04783, K02854, K00124, K05838, K11179, K09159, K06039, K05527, K03673, K07121, K03808, K04013, K04016, K07674, K07685, K09909, K03185, K01139, K13643, K04017, K03417, K13888, K14187, K06980, K17870, K10804, K01792, K12060, K03071, K06190, K12057, K12062, K12072, K12368, K10110, K12369, K12370, K12371, K12372, K07712, K15536, K03184, K00885, K03078, K03477, K09900, K02506, K01175, K01766, K02344, K02742, K03591, K03632, K03633, K03645, K03674, K03764, K03804, K05809, K06205, K06866, K06899, K08990, K09896, K09898, K09901, K09904, K09908, K09910, K11258, K18800, K19226, K08992, K07821, K09894, K00242, K07236, K07237, K06916, K16868, K09890, K03603, K03777, K02569, K01407, K05790, K03835, K08306, K01096, K04753, K07227, K02681, K07186, K10000, K09906, K01525, K03212, K07640, K04755, K09997, K09917, K09781, K06149, K11740, K02430, K09802, K01141, K07286, K11747, K07340, K09160, K03893, K12139, K02363, K03746, K02553, K05589, K09471, K04744, K13771, K07323, K04062, K03304, K02386, K02393, K02394, K07054, K13498, K03583, K07246, K04085, K03580, K02427, K03641, K12661, K03562, K02192, K05787, K11734, K13637, K00568, K06175, K02483, K13818, K02197, K02195, K02194, K05539, K00324, K02552, K07665, K02549, K00228, K07312, K15830, K15832, K12136, K06165, K07310, K12143, K12507, K02532, K18446, K03762, K07664, K19222, K00164, K18130, K19340, K19341, K14977, K07792, K00972, K16214, K07008, K07497, K01582, K02841, K01664, K03274, K04656, K09800, K09766, K02413, K02404, K06603, K02407, K04562, K06221, K01494, K07182, K02398, K09770, K02396, K02843, K04061, K01496, K08316, K02411, K02409, K01226, K03767, K03271, K01673, K06182, K03610, K05797, K11382, K18291, K19212, K03195, K18955, K07288, K13795, K16293, K16294 |
| FC:50ppm |  |  |  |
| FC:150ppm | K19052 |  | K01561, K13923, K03343, K01236, K06044, K06985 |
